# Synthesis of anti-depressant molecules *via* metal-catalyzed reactions: a review

**DOI:** 10.1039/d3ra06391g

**Published:** 2024-02-26

**Authors:** Aqsa Kanwal, Uzma Afzal, Muhammad Zubair, Muhammad Imran, Nasir Rasool

**Affiliations:** a Department of Chemistry, Government College University Faisalabad 38000 Pakistan aqsa6373@gmail.com uzmaafzal520@gmail.com zubairmkn@gcuf.edu.pk nasirrasool@gcuf.edu.pk +92-3085448384; b Chemistry Department, Faculty of Science, King Khalid University P.O. Box 9004 Abha 61413 Saudi Arabia imranchemist@gmail.com

## Abstract

Depression is one of the most mutilating conditions in the world today. It has been difficult to make advancements toward better, more effective therapies since the introduction of antidepressant medicines in the late 1950s. One important field of medicinal chemistry is the synthesis of antidepressant molecules through metal-catalyzed procedures. The important role that different transition metals, including iron, nickel, ruthenium, and others, serve as catalysts in the synthesis of antidepressants is examined in this review. Key structural motifs included in antidepressant drugs such as tricyclic antidepressants (TCAs), selective serotonin reuptake inhibitors (SSRIs), and others can be synthesized in a variety of effective ways using metal-catalyzed steps. This review examines current developments in the catalytic synthesis of antidepressants and their potential application over the previous thirteen years.

## Abstract 2

Depression is one of the most mutilating conditions in the world today.

## Graphical abstract

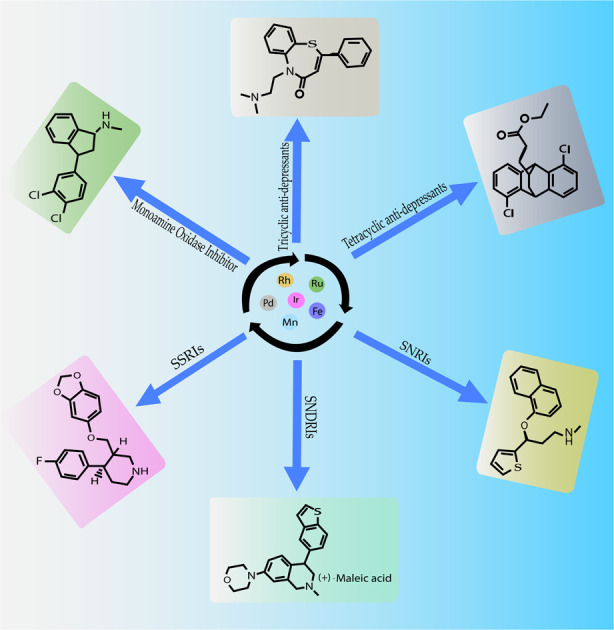

Depression, the third major global health concern, is anticipated to escalate to the second most significant health challenge worldwide by 2030^1–3^ According to WHO, 3.8% of the world's population is affected by depression; this includes 5% of adults (4% of men and 6% of women) and 5.7% of persons sixty years of age and over. The prevalence of depression affects roughly 280 million people worldwide. A 2011 survey conducted by the World Mental Health Survey across 17 countries revealed that one in 20 individuals went through a depressive episode. Depression can inflict significant distress, resulting in disability and even death for the affected individual. On a global scale, an estimated 700 000 annual suicide deaths are linked to depression.^4,5^ Depressive disorders still have a restricted range of treatments.^6^ Thus, it is particularly essential to develop novel antidepressants that have a quick onset, low side effects, with enhanced cognitive function. A significant area of study in the discipline is the development of novel dual- or multi-target antidepressants.^7–9^

Antidepressants have shown effectiveness in alleviating symptoms and enhancing the quality of life for individuals with moderate to severe depression. Approximately 50–60% of people with depression experience substantial improvement when using these medications.^10^ Depression is a common mood syndrome triggered by the improper release of monoamine neurotransmitters as noradrenaline, dopamine also serotonin in the CNS with the malfunction of noradrenergic, dopaminergic, and serotonergic systems.^11–14^

Anti-depressants are psychotropic drugs, primarily utilized to treat mental diseases characterized by depressed mood. They also can reduce nervousness, somatic symptoms, and anxiety. Tricyclic antidepressants (amitriptyline, imipramine, and nortriptyline), as well as oxidase inhibitors (such as moclobemide and phenelzine), and SSRIs, (such as citalopram, fluoxetine, and paroxetine), as well as SNRIs, (such as reboxetine), and reuptake inhibitors of serotonin–norepinephrine (desvenlafaxine and venlafaxine),^15,16^ and herbal remedies (St. John's Wort),^17^ tetracyclic antidepressants (mirtazapine) are all types of antidepressant medications that raise the levels of several monoamines in the synaptic clefts.^18^ Here's a concise way to summarize in Table 1.

**Table 1:** List of FDA-approved drugs

Classification of antidepressants	FDA-approved antidepressant drugs	Mechanism of action	References
Selective serotonin reuptake inhibitors (SSRIs)	Paroxetine, fluoxetine, sertraline, escitalopram	SSRIs, function by impeding the serotonin reuptake in the brain. By inhibiting the serotonin transporters, SSRIs prolong the presence of serotonin in the synaptic space between neurons. This prolonged serotonin activity enhances its effects on mood regulation and neuronal communication, potentially mitigating symptoms of depression and related mood disorders	19 and 20
Serotonin-norepinephrine reuptake inhibitors (SNRIs)	Milnacipran, venlafaxine, duloxetine, reboxetine	SNRIs, work by blocking the reuptake of both serotonin and norepinephrine in the brain. By inhibiting the transporters responsible for reabsorbing these neurotransmitters, SNRIs increase their availability in the synaptic space between neurons	21 and 22
Tricyclic antidepressant	Aripiprazole, desipramine, clomipramine	TCAs raise the levels of these neurotransmitters in the brain and prevent neurons from reabsorbing 5HT and NA by axonal absorption. These drugs are generally used for people suffering from migraine and chronic pain	23 and 24
Monoamine oxidase inhibitors	Toloxatone, diclofensine, selegiline	MAO inhibitors prevent the breakdown of serotonin, dopamine, and norepinephrine by binding to monoamine oxidase, elevating their levels in nerve endings. This action sustains these key neurotransmitters, potentially aiding antidepressant effects by prolonging their activity in the central nervous system	25 and 26
Atypical antidepressants	Rolipram, vilazodone, vortioxetine, lumateperone, agomelatine	These medications target different neurotransmitter systems, such as dopamine, norepinephrine, and serotonin, but their exact mechanisms can differ	27

Antidepressants work through a variety of key receptors and neurotransmitter systems: SSRIs and some SNRIs primarily boost serotonin levels by affecting 5-HT receptors; others like SNRIs and TCAs target norepinephrine, impacting adrenergic receptors (Fig. 1).^28^

**Fig. 1:**
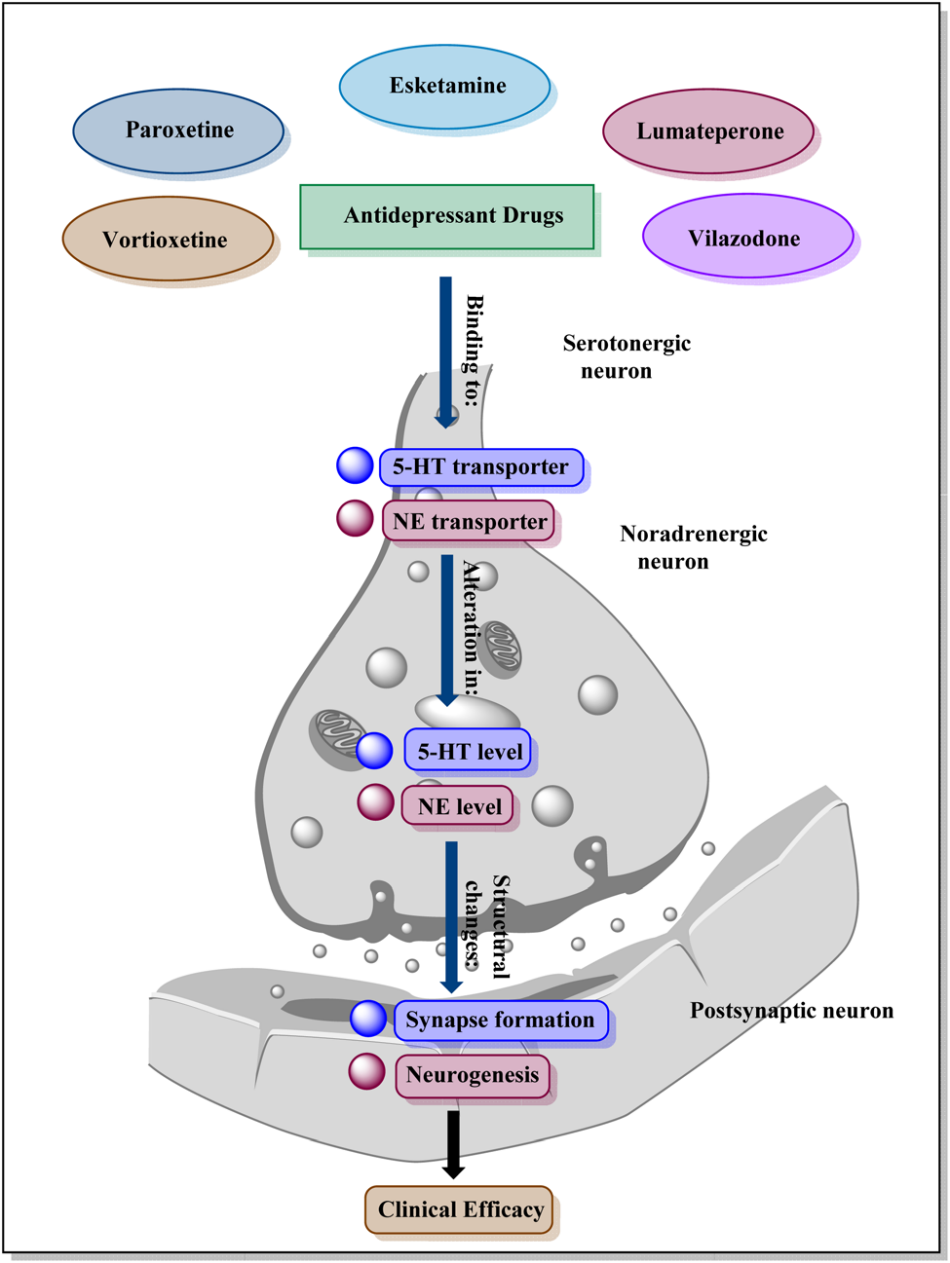
General mechanism of action of antidepressants.

Atypical antidepressants like bupropion focus on dopamine reuptake. Ketamine influences glutamate receptors, particularly NMDA receptors, for its rapid-acting effect. While not directly targeted, GABA and cannabinoid receptors may be indirectly affected by antidepressants. Antidepressants often impact BDNF levels, influencing neuroplasticity and neuronal survival through TrkB receptors.^29^ These receptors and associated neurotransmitter systems are targeted by various classes of antidepressants, collectively impacting mood, emotions, and brain function to alleviate symptoms of depression.^30,31^

Metal-catalyzed transformations have been employed in the synthesis of antidepressants at several sites along the pathway, leading to the development of C–C, and C–N bonds and the functionalization of aromatic rings.^32^ In particular, SSRIs, including sertraline and fluoxetine, which are extensively used to treat depression, have been synthesized *via* couplings catalyzed by palladium.^16,33^

Moreover, the synthesis of other kinds of antidepressants, MO inhibitors, and tricyclic antidepressants has also been accomplished *via* metal-catalyzed reactions.^34^ These crucial compounds have been synthesized in vast quantities owing to the development of more effective and environmentally friendly synthetic pathways, which were greatly facilitated by the use of metal catalysts.^35^

Metal-mediated reactions have become integral in drug synthesis due to their adaptability, selectivity, mild reaction conditions, and compatibility with complex molecules, contributing significantly to the pharmaceutical industry's synthetic capabilities.^36–40^

This review provides a comprehensive analysis of synthetic pathways for a range of metal-catalyzed antidepressants, commercially available medications, and bioactive compounds with antidepressant properties. It offers valuable insights for synthetic chemists and pharmacists, elucidating the utilization of various metals and their complexes across different methodologies. By encompassing a broad spectrum of compounds, this review aims to enhance understanding within the field, serving as a guide for future chemists seeking to leverage these methodologies effectively.

## 1. Ruthenium-catalyzed reactions

Selegiline when employed with L-DOPA is a highly successful treatment for both Parkinson's along Alzheimer's disease. It is a monoamine oxidase-B (MOB) antagonist that is specific and irreversible.^41,42^ Independent of MAO inhibitors, the propargylamine pharmacophore of selegiline and analogous drugs also seems to possess neuroprotective effects.^43^

**Scheme 1:**
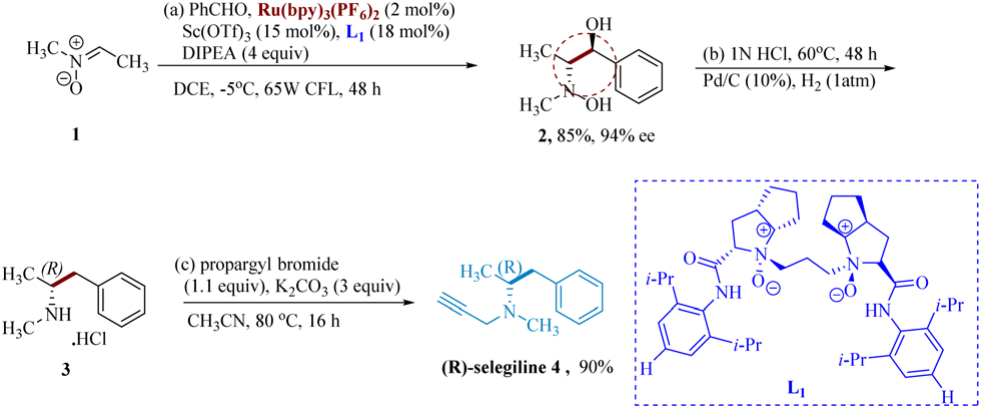
Synthesis of *N*-methyl-(phenyl propan 2-yl)prop-2-yn amine (selegiline).

Ye *et al.* undertook the synthesis of selegiline using Ru photocatalyst & chiral *N*,*N*′-dioxide coordinated unique earth ion L_1_ which work synergistically to initiate the photocatalytic enantio-selective reductive coupling of aromatic aldehydes with nitrones. The asymmetric radical formation is sparked by chiral Lewis acid, which serves as a crucial framework for assembling the essential precursor and produces enantiopure vicinal hydroxyl amino alcohols in good to outstanding yields exhibiting great stereo-selectivity. Here, Sc(OTf)_3_ serves as the Lewis acid & Ru(bpy)_3_(PF6)_2_ (photocatalyst). Asymmetric reductive coupling of benzaldehyde with nitrone 1 gave the product 2 as the key diastereomer 11/1 dr with 94% enantiomeric excess (ee). Additionally, ®-methamphetamine hydrochloride 3 was produced by dehydroxylating vicinal hydroxyamino alcohol 2 in an aq. HCl at moderate Pd/C-catalyzed hydrogenolysis. Crude 3 was *N*-propargylated with K_2_CO_3_ in acetonitrile to get (−)-selegiline 4 (Scheme 1).

**Fig. 2:**
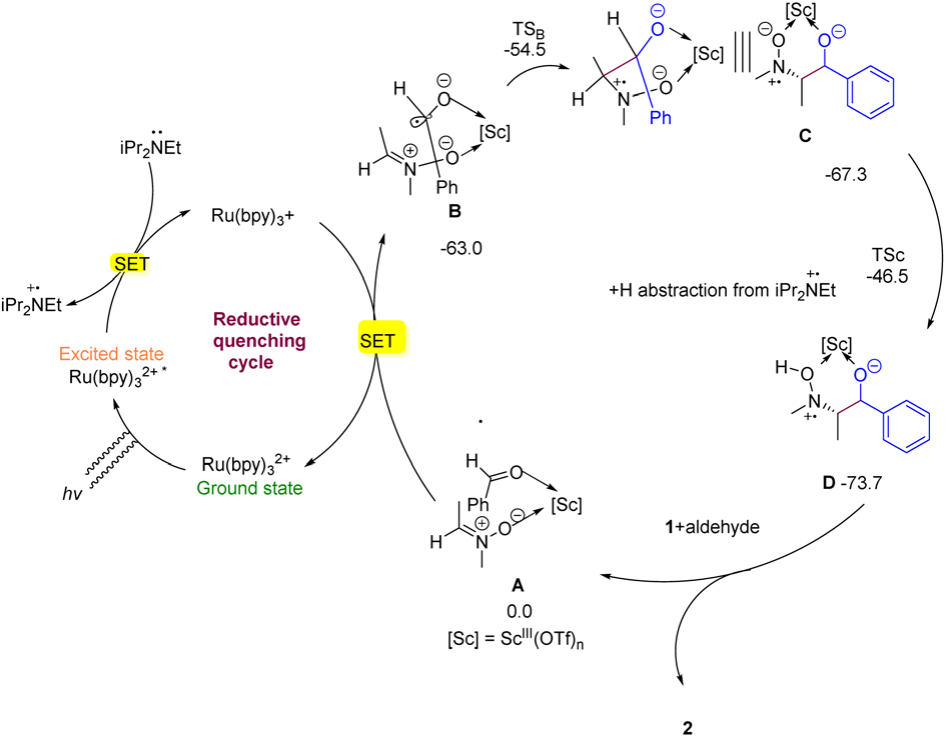
Mechanism for synthesis of selegiline.

After Ru(bpy)_3_^2+^ is photoexcited and reductively quenched by DIPEA, [iPr_2_(Et)N˙]^+^ and Ru(bpy)^3+^ (E1/2II/I = 1.33 V *vs.* SCE in MeCN) are generated. This is sufficiently to reduce complex A *via* intermolecular SET (onset potential *E*_op_ > −0.5 V *vs.* SCE) and yield the radical complex B. Indeed, DFT calculations confirmed that the electron affinity of A is much higher (∼63.0 kcal mol^−1^ in free energy) than that of nitrone 1 (∼23.4 kcal mol^−1^) and 4-fluorobenzaldehyde (∼45.1 kcal mol^−1^) in solvent, and the as-generated cross-coupling precursor B has spin density localized predominantly on the aldehyde moiety. Subsequently, N-radical intermediate C (or C′ of *anti*-configuration) is formed through an analogous 6-*endo-trig* radical annulation, and the transition state TS_B_ leading to a *syn*-configuration is predicted to be by 1.9 kcal mol^−1^ favored over the *anti*-configuration transition state TS_B′_, C upon hydrogen abstraction from [iPr_2_(Et)N˙]^+^ affords regioselectively intermediate D (*via*TS_C_) other than D′ (*via*TS_C′_). Finally, protonation of D gives the desired vicinal hydroxyamino alcohol 2 as a major diastereomer. Moreover, DFT calculations also showed that the formation of cross-coupling precursor B is overwhelmingly favored over the formation of homocoupling precursors, accounting well for the reaction specificity towards cross-coupling rather than homocoupling. Based on this mechanism, the diastereoselectivity of vicinal hydroxyamino alcohols, such as 2, can be analyzed by comparing the energy of the six-member ring transition state TS_B_ with that of TS_B′_. Chiral scandium complex I, which involves a Re-to-Re-facial assault of the ketyl radical to nitrone 1, exhibits enantioselectivity (Fig. 2).^44,45^

**Scheme 2:**
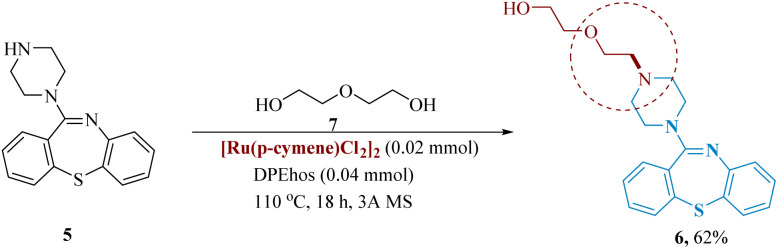
Synthesis of 2-(2-(4-(dibenzo[1,4]thiazepin-11-yl)piperazin-1-yl)ethoxy)ethan-1-ol (quetiapine).

In medicinal chemistry, the polyethylene glycol scaffold has gained much significance. Rossi *et al.* described the hydrogen borrowing reductive amination method of PEG functionalization of amines was described. This was achieved by reacting the phosphorus-containing dppf or DPE with the catalyst [Ru(*p*-cymene)Cl_2_]_2_ to produce a range of 1° and 2° amine products. They were able to directly produce quetiapine 6 from 11-(piperazine-1-yl)-dibenzo[*b*,*f*][1,4]thiazepine 5 in 62% isolated yield (Scheme 2).^46^

Recently, ketamine & its (*S*)-enantiomer, esketamine, were investigated for their immediate anti-depressant effects and have been proposed as a potential medication for depressive disorder, as well as resistant depression.^47^*In vitro*, (*S*)-ketamine (esketamine) has a 3–4 fold higher affinity than (*R*)-ketamine for the glutamate *N*-methyl d-aspartate receptor.^48^ Esketamine has attracted more interest in the advancement of an antidepressant drug in short-term treatment.^49,50^ Chen & Lu synthesized Ketamine which primarily functions as a non-competitive NMDA receptor antagonist.^51,52^

**Scheme 3:**
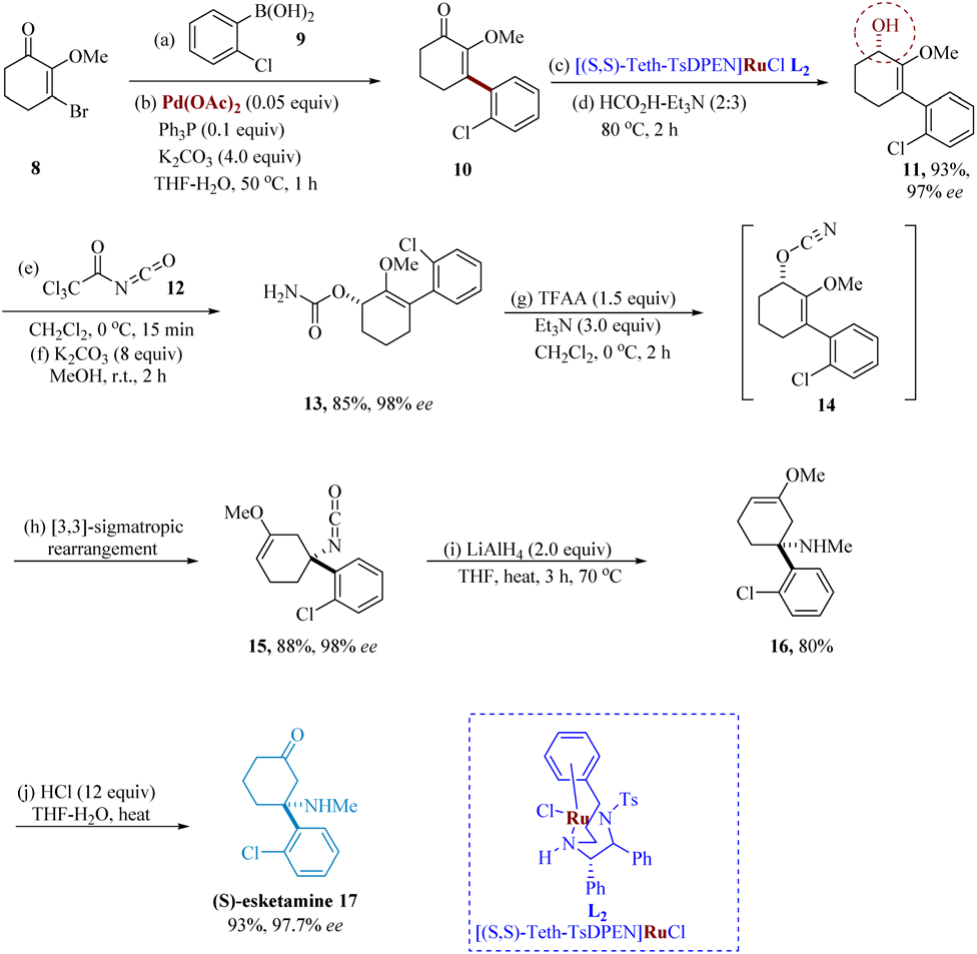
Synthesis of 3-(2-chloro-phenyl)-3-(methyl-amino)cyclohexane-1-one (esketamine).

The Noyori catalytic AH of enone 10 was used to set up the stereogenic center in 11 and the [3,3]-sigmatropic re-arrangement of the allylic cyanate intermediate 14 to generate the quaternary stereogenic center in isocyanate 15 with exceptional stereochemical relay were two features of the small-scale asymmetric synthesis of esketamine. For the asymmetric reduction of enone 10, several ruthenium-based catalysts were investigated; however, at 0.1% loading, only [(*S*,*S*)-Teth-TsDPEN] RuCl afforded full conversion in 97–98% ee (Scheme 3).^53,54^

Other pharmaceutically useful substances, NK 1-receptor antagonist, antifungal amorofine, and SCH50911 GABA-antagonist, contain reboxetine, which is a SNRI.^55^

**Scheme 4:**
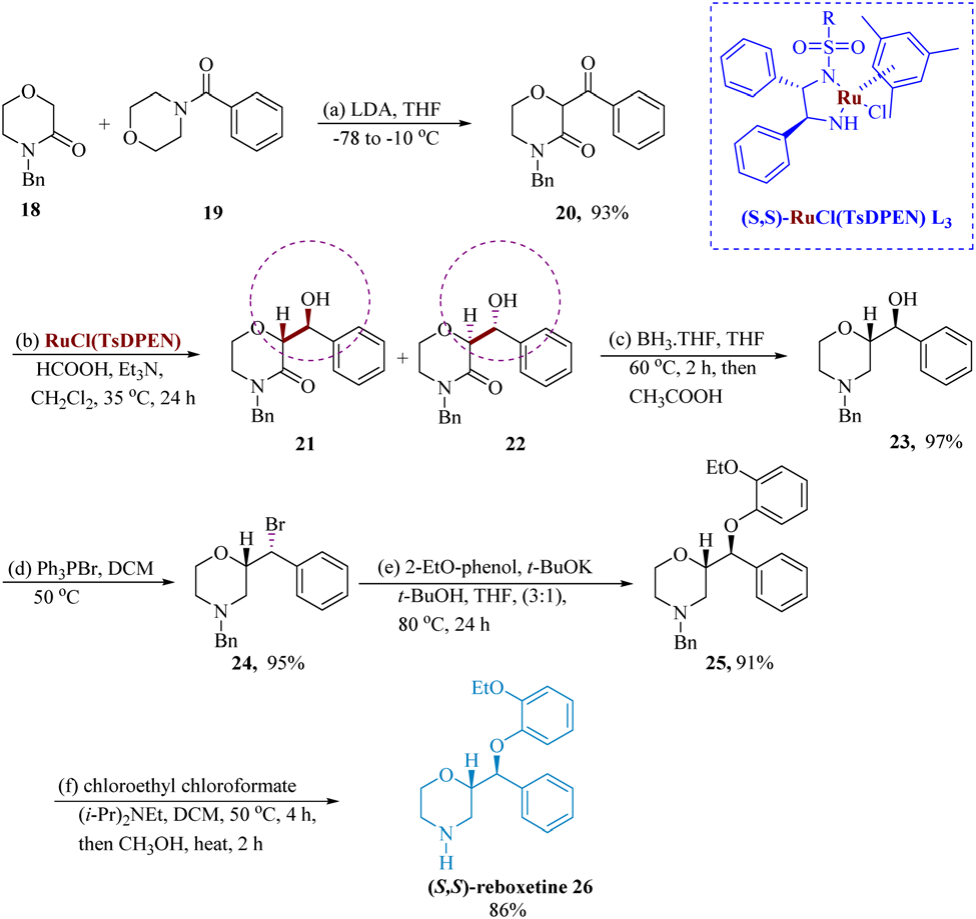
Synthesis of 2-(2-ethoxy phenoxy)(phenyl)methyl morpholine (reboxetine).

Son & Lee developed the dynamic kinetic resolution-mediated asymmetric transfer hydrogenation (ATH) of 2-benzoyl morpholine-3-ones served as a crucial step in the stereoselective synthesis of reboxetine 26. With a 93% yield, the *N*-benzyl-2-aroylmorpholin-3-one 20 was produced when the *N*-benzyl-3-morpholinone 18 was condensed with *N*-aroylmorpholines 19 in the presence of LDA. The alcohols (2*R*,3*S*)-21 and (2*S*,3*R*)-22 were produced in a combined yield of 90% by the ATH reaction of 20 with catalyst (*S*,*S*)–RuCl(TsDPEN) L_3_, which was mediated by dynamic kinetic resolution. After being reduced by BH_3_ THF, the lactam 21 produced the corresponding morpholine benzyl alcohol 23 in 97% yield, which was then processed by Ph_3_PBr_2_ to produce the respective morpholine bromide derivatives 24 in 95% yield. In the presence of *t*-BuOK, molecule 24 underwent bromide displacement with 2-ethoxyphenol to generate the *N*-benzyl-protected derivatives 25 91% of the time. After being treated with chloroethyl chloroformate and methanolysis, the compound 25 synthesized the target molecule (*S*,*S*)-reboxetine 26 with an 86% yield (Scheme 4).^56^

**Scheme 5:**
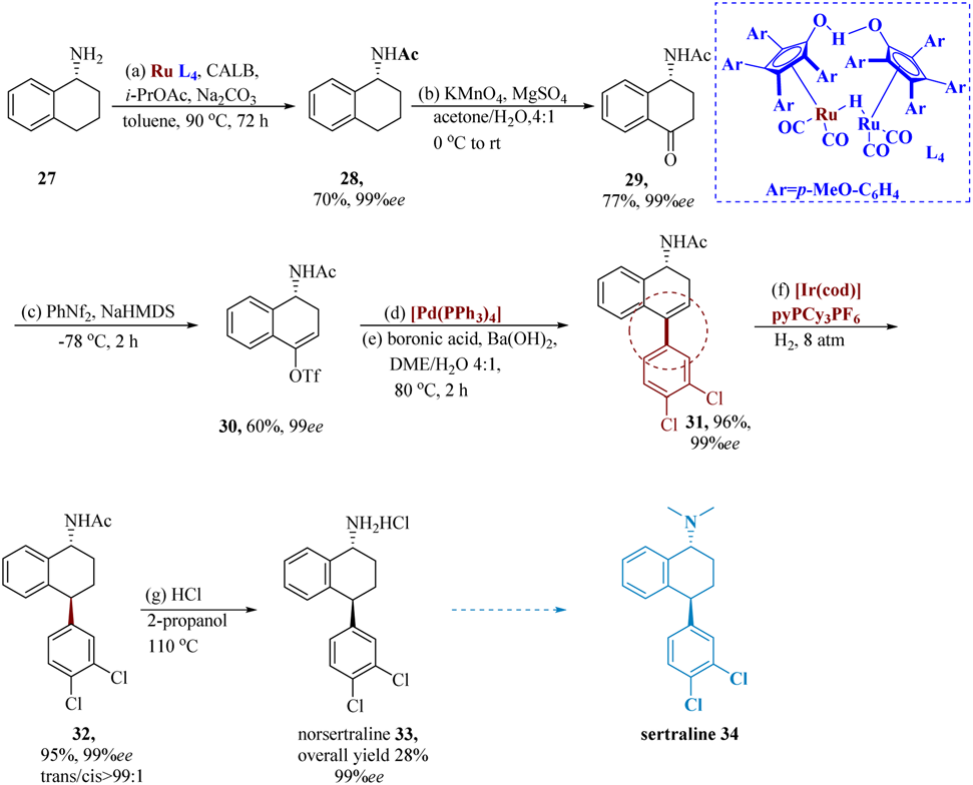
Synthesis of 4-(3,4-dichlorophenyl)-1,2,3,4-tetra hydro-naphthalene amine hydrochloride (norsertraline).

An SSRI antidepressant, nor-sertraline is a sertraline analog. Thalen *et al.* developed a new pathway to 33 using CALB, isopropyl acetate, and Na_2_CO_3_ in toluene and readily available 1, 2, 3, and 4-tetrahydro-1-naphthyl amine 27 and DKR of primary amines was developed. To start, the standard procedure was used to apply DKR to achieve 28 in 70% yield and 99% ee. KMnO_4_ had an impact on oxidation at the C-4 position to produce 29. Following the formation of the enolate and its trapping with *N*-phenyl-bis(trifluoromethanesulfonimide), the resultant compound 30, reacted with 3, 4-dichlorophenyl boronic acid to generate 31 with a 96% yield and 99% ee. It was possible to obtain 32 in 95% with a *trans*/*cis* ratio of >99 : 1 using trans selectivity hydrogenation with the Crabtree catalyst. Following the deprotection of the acetamide in an acidic environment, nor-sertraline 33 produced a yield of 95% with full retention of dr and ee (99% ee, and *trans*/*cis* >99 : 1) (Scheme 5).^57^

**Scheme 6:**
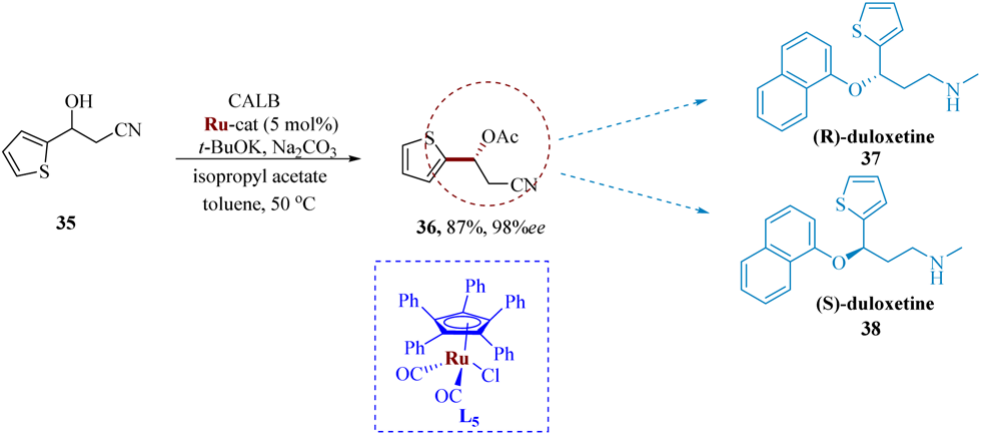
Synthesis of *N*-methyl-3-(naphthalen-1-yloxy)-3-(thiophen-2-yl)propane amine (duloxetine).

Träff *et al.* reported the lipase-catalyzation *via* kinetic resolution of a racemic β-hydroxy nitrile leading to the stereo-inversion to produce the eutomer (*S*)-duloxetine through Mitsunobu yields an enantiopure *R* diastereomer of duloxetine.^58^ By using the DKR process, the yields were significantly improved. Candida antarctica lipase as well as ruthenium catalyst (Shvo's catalyst) L_5_, were employed in the DKR of the starting molecule β-hydroxy nitrile 35 to produce the analogous β-cyano acetate 36 in yield of 87% & 98% ee. The production of both (*R*)-37 as well as (*S*)-duloxetine 38 was made possible by subsequent synthetic procedures (Scheme 6).^59^

## 2. Iron-catalyzed reactions

**Scheme 7:**
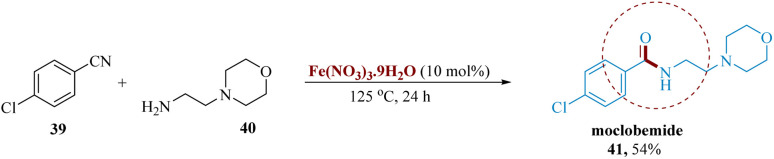
Synthesis of 4-chloro-*N*-(2-morpholinoethyl)benzamidine (moclobemide).

Allen *et al.* reported the synthesis of moclobemide 41 using Fe (NO_3_)_3_·9H_2_O, a low-cost catalyst, in a modest isolated yield by using readily available starting materials such as nitrile 39 and amine 40. When primary unbranched amines combine with nitriles that are not excessively electron-rich, the reaction is most favoured (Scheme 7).^60^

Chopadea *et al.* reported an effective and convenient procedure for the Michael addition using Fe(acac)_3_ (5 mol%) as an effective catalyst, which catalyzes the Michael addition reaction of nitromethane to chalcone to produce corresponding γ-nitroketone derivatives with good yields under milder conditions.

**Scheme 8:**
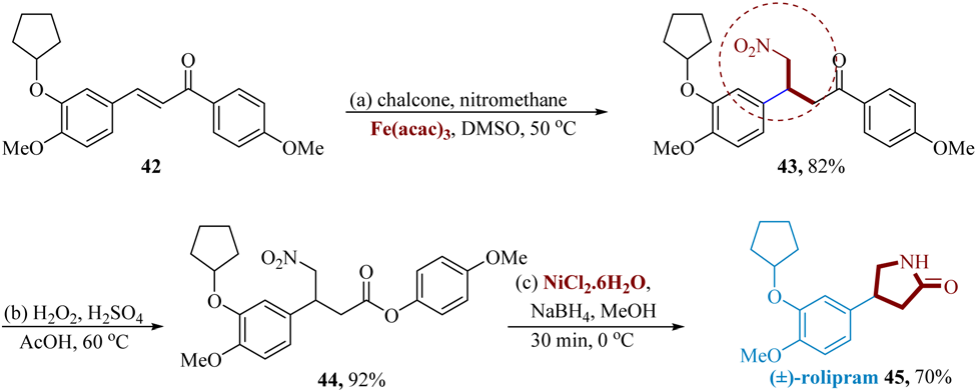
Synthesis of 4-(3-(cyclo-pentyloxy)-4-methoxy phenyl)pyrrolidin-2-one (rolipram).

Chalcone 42 as the starting material was added to iron-catalyzed Fe (acac)_3_ (5 mol%), and Michael's addition of nitromethane produced γ-nitro ketone 43 in 82% of the reactions. The Baeyer–Villiger reaction 43 using H_2_O_2_ in AcOH provided the analogous γ-nitro ester 44. Following a Ni-catalyzed reduction of the NO_2_ using sodium borohydride in combination with nickel chloride to generate cyclic amide as (±)-rolipram 45 in 3 steps with a total yield of 52.16%. (±)-Rolipram 45 acts as a neurotransmitter inhibitor drug molecule (Scheme 8).^61,62^

## 3. Nickel-catalyzed reactions

**Scheme 9:**
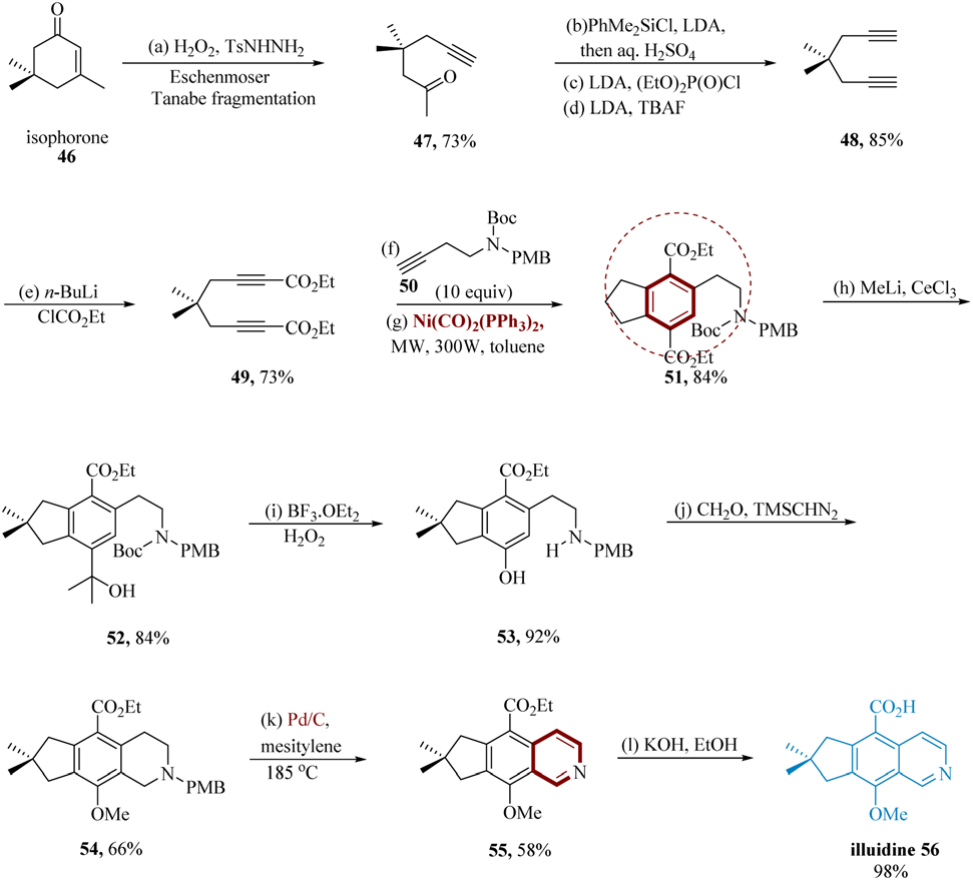
Synthesis of 9-methoxy-7,7-dimethyl-7,8-di-hydro-6*H*-cyclo penta iso-quinoline-5-carboxylic acid (illuidine).

Illudalic acid is “the first potent, selective” MAOI, with an IC_50_ value of 18 ± 7.1 M in initial testing. Gaston *et al.* reported the alkaloid network that enables the formation of illudalic acid is illudinine. The synthesis of illudinine begins with known diyne 48, produced from isophorone 46 that underwent Eschenmoser–Tanabe fragmentation, was subsequently lithiated at a terminal (≡)-bonds and carboxylated to diester 49. Under MW irradiation in C_6_H_5_CH_3_ for two minutes at a power of 300 W, this molecule 49 was directed with alkyne 50*via* a Ni(CO)_2_ (PPh_3_)_2_-catalyzed [2 + 2 + 2] cyclo-trimerization. The alkyne 50 was chosen as the PMB group may be effectively eradicated in an oxidation step to complete the whole synthesis. The cyclotrimerization product 51 was separated in an 84% yield. The phenolic OH was effectively introduced at C-7 in the subsequent steps. Remarkably, the 3° alcohol 52 was the single product produced at 84% when 51 was treated with an excess of CH_3_Li in CeCl_3_. Treatment with BF_3_OEt_2_/H_2_O_2_ in DCM at 0 °C mediates the successive carbenium ion rearrangement of 52 to 53. A 92% yield of phenol 53 is produced when the Boc group is simultaneously removed, and this prepares the way for the traditional Pictet–Spengler reaction to assemble the tricyclic skeleton. The corresponding tetrahydro iso-quinoline is produced by treating 53 with formaldehyde along with a sodium acetate buffer, and it is then immediately transformed to the methyl ether 54, treated with trimethylsilyl diazomethane (66% over two steps). Pd/C in mesitylene was used to oxidize 54 to iso-quinoline 55 at a temperature of 185 °C. In a combined yield of 58%, these conditions result in the simultaneous exclusion of the PMB-protecting group. This extremely selective and converged total synthesis of illudinine 56 is finished by quantitatively saponifying the ester by 40% aq. KOH in EtOH/H_2_O has a ratio of (95 : 5) (Scheme 9).^63^

Serotonin reuptake and other anxiety-related illnesses are specifically blocked by sertraline hydrochloride.^64^ Sertraline 145 has one or more asymmetric centers, and as a result, the naturally dynamic 1*S*,4*S*-enantiomer, and sertraline, must be produced with great optical purity.

**Scheme 10:**
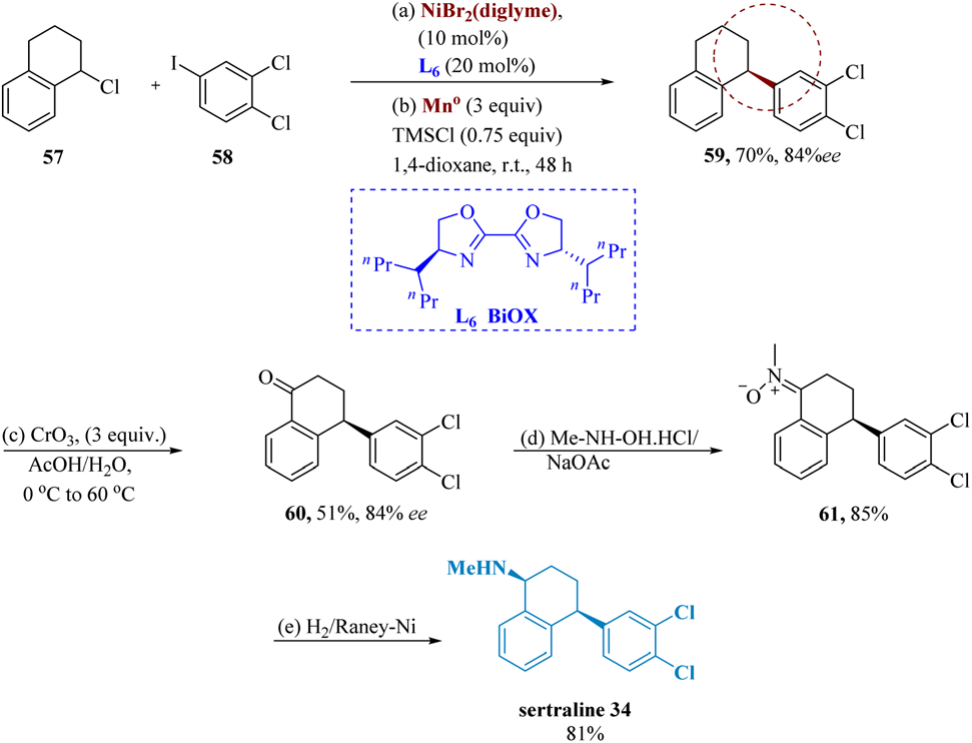
Synthesis of 4-(3,4-dichlorophenyl)-*N*-methyl-1,2,3,4-tetrahydro naphthalene amine (sertraline).

Poremba *et al.* developed Ni-catalyzed enantio-selective reductive coupling that produces 1,1-diarylalkanes with increased yields and enantioselectivity, using 4-heptyl-BiOX L_6_. Chiral tetrahydronaphthalene 149 is produced in 70% yield and 84% ee by cross-coupling 1-chloro-1,2,3,4-tetrahydro-naphthalene 57 with widely accessible iodobenzene 58. Tetralone 60 was produced in 51% yield by the benzylic oxidation of 59 utilizing CrO_3_ having 3 equiv. in AcOH/H_2_O. Tetralone 60 and *N*-methyl hydroxylamine are condensed to form nitrone 61, whose reduction yields the required amines (sertraline) 34 (Scheme 10).^65^

**Scheme 11:**
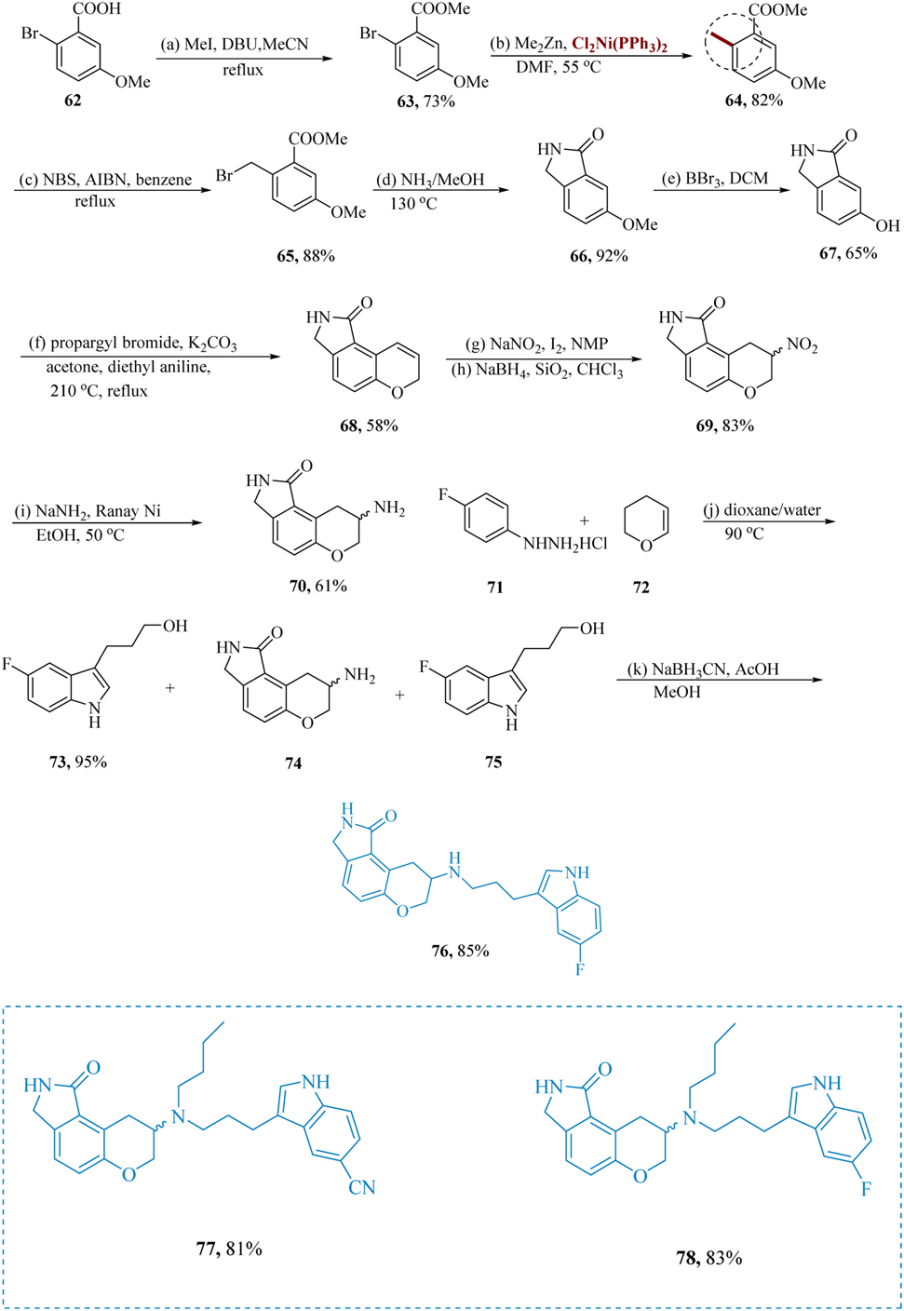
Synthesis of 8-((3-(1*H*-indol-3-yl)propyl)amino)-2,3,8,9-tetrahydro-pyrano[3,2-*e*]isoindol-1(7*H*)-one.

Shen *et al.* described new lactam-fused chroman compounds with dual affinities for the 5-HT1A as well as the serotonin transporter.^66,67^ An effective pathway to intermediates 66 was necessary for the formation of des-fluoro lactam-chroman amines 76. Under the specified conditions, the aryl halide 63 was converted to the toluene derivative 64 at the start of the reaction. Aryl bromide and alkyl zinc undergo a cross-coupling that is accelerated by a transition metal. Bis-(triphenylphosphine) nickel(ii) dichloride catalyzed the reaction of 63 at 50 °C using dimethylzinc in DMF, resulting in 64. The production of the five-membered lactam 66, which results in the regioselective isomer 74, was eventually made possible by the production of 64. Reductive amination was the strategy they had in mind for the synthesis of indoles to produce the necessary end products. The penultimate secondary amines 76 of new lactam-fused chroman compounds, primarily compound 77/78 having dual affinity at the 5-HT1A as well as the serotonin transporter *in vitro* cAMP turnover model, were produced by reductive amination of lactam-fused chroman amines *via* indole-substituted alcohols 75 (Scheme 11).^68^

**Scheme 12:**
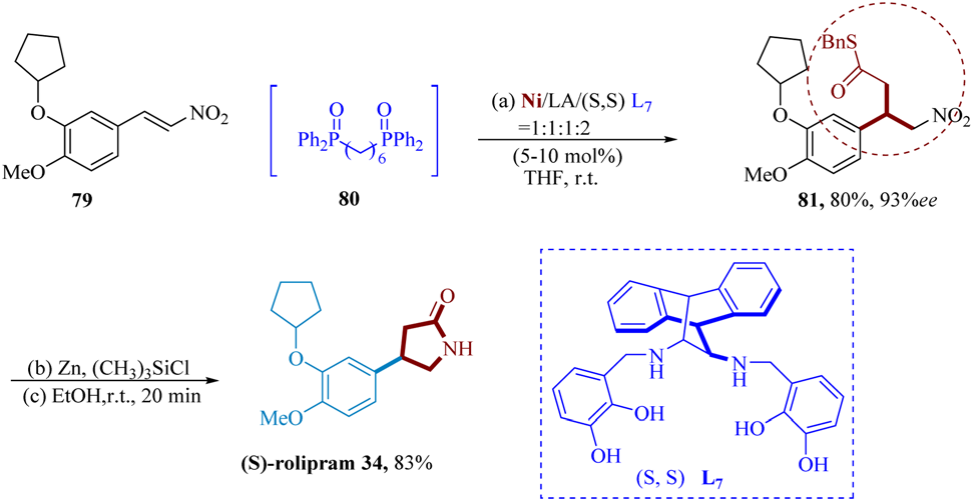
Synthesis of rolipram.

Furutachi *et al.* reported A hetero-bimetallic Ni/La-salan 2d complex of phosphine oxide was used in the catalytic decarboxylative 1,4-addition to 4-MeO-3-cyclopentyloxyC_6_H_3_-substituted nitro-alkene 79, which produced product 81 in 80% yield and 93% ee. By treating 81's nitro group with Zn and (CH_3_)_3_SiCl, the nitro group was converted to an amine, and subsequent cyclization occurred during workup to produce (*S*)-rolipram 34 in yield of 83% (Scheme 12).^69^

Bioactive compounds, pharmaceuticals, and organic functionalized materials all frequently contain C(sp_2_)–S bonds.^70–75^ As a result, metal-catalyzed C(sp_2_)–S formation is now receiving more and more attention.^76–79^ The direct C(sp_2_)–H thiolation of amides and sulfides was effectively used as the crucial step in the formation of quetiapine.

**Scheme 13:**
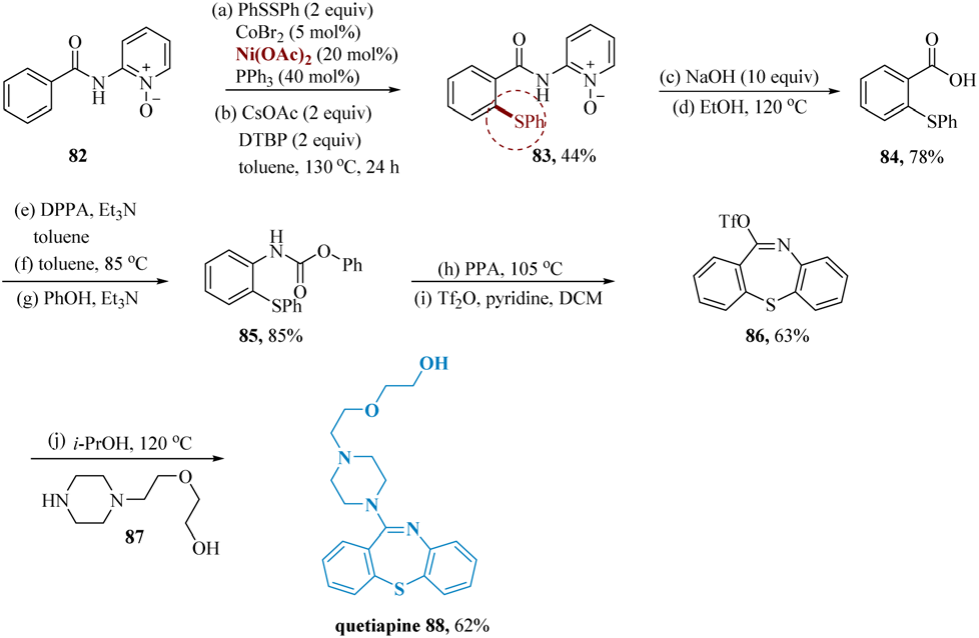
Synthesis of 2-(2-(4-(dibenzo[1,4]thiazepin-11-yl)piperazin-1-yl)ethoxy)ethan-1-ol (quetiapine).

Li & wang reported the synthesis of quetiapine an atypical antipsychotic drug that has been licensed for treating bipolar disorder and schizophrenia.^80,81^ For accessing quetiapine this synthetic approach was constructed using direct C–H thiolation of benzamides as opposed to the conventional synthetic procedure, which involved cross-coupling of benzenethiols and aryl halides.^80,82,83^ The anticipated product 83 on a gram scale was produced by thiolating 1,2-diphenyldisulfane with 2-benzamidopyridine-1-oxide 82. Then, by hydrolyzing the amide, a derivative of benzoic acid 84 was produced. After that, the compound 84 underwent Curtius rearrangement and was treated with phenols to produce carbamate 85. After that, polyphosphoric acid facilitated cyclization, which was then followed by pseudohalogenation to produce triflate 86. Finally, the nucleophilic substitution of the piperazine 87 with triflate 86 led to the desired Quetiapine 88 product (Scheme 13).

**Fig. 3:**
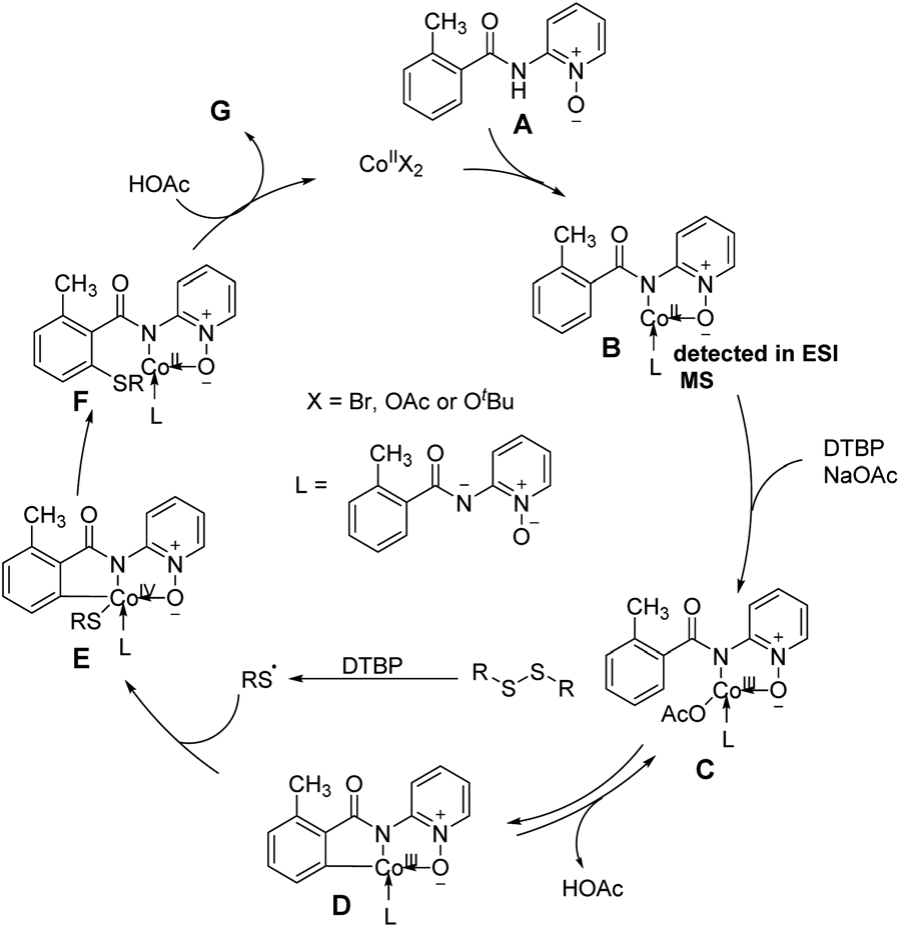
Mechanism for synthesis of quetiapine.

Based on the above-mentioned mechanistic investigations a proposed mechanism is illustrated in Fig. 3. Initially, the coordination of benzamide A with a cobalt(ii) catalyst and a subsequent ligand exchange brought intermediate B, which was detected by ESI-MS. Next, the cobalt(ii) complex is oxidized by DTBP yield cobalt(iii) intermediate C, which undergoes a reversible C–H cobaltation to afford cobaltacycle species D. Subsequently, radical coupling of intermediate D with the thioether radical yielded from disulfides in the presence of DTBP provides cobalt(iv) intermediate E. Finally, reductive elimination of E following protonation furnishes the desired product G and cobalt(ii) catalyst to finalized the catalytic cycle. The rate-determining progression could be the reductive elimination of intermediate E (Fig. 3).^83^

## 4. Palladium catalyzed reactions

Indatraline is a potential psychoactive complex with significant binding as well as inhibitory activity aimed at monoamine reuptake neuronal sites, together with serotonin transporters & dopamine.^84,85^ Behavioral assays and *in vivo*, dialysis have suggested that indatraline has a strong dopaminergic mode of action with a prolonged half-life.^86,87^ Additionally, indatraline was found to decrease the self-administration of cocaine in monkey trials.^88^

**Scheme 14:**
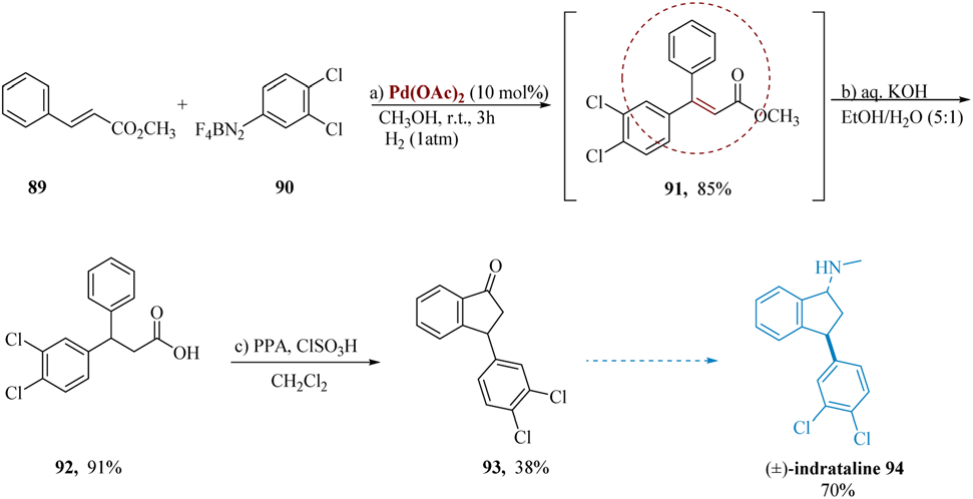
Synthesis of 3-(3,4-di-chlorophenyl)*N*-methyl-2,3-di-hydro-1*H*-inden amine (indatraline).

Pastre & Correia developed a synthesis of the psychoactive drug indatraline. To get a saturated β,β-di arylated product 91 with 85% yield, methyl cinnamate 89 was treated to Heck arylation with 3,4-dichloro benzene-diazonium tetra-fluoroborate 90, as it was accompanied through an *in situ* catalytic hydrogenation of adduct 91. Due to the generation of side products during the dehalogenation process, control of a hydrogenation phase is important. Next, the ester 91 was hydrolysed through aqueous KOH to produce the analogous acid 92 in a 91% yield. Successive cyclization *via* PPA and/or ClSO_3_H produced the well-known intermediate 93 of (±)-indatraline 94 in yields of 38% & 70% (Scheme 14).^89,90^

2-Oxazolidinones have attracted considerable attention owing to their significant heterocyclic scaffolds having a diverse range of pharmacological activities. Oxazolidinones are a significant class, naturally existing substances and promising medicinal frameworks with a variety of biological and pharmacological activities, including antimicrobial, antibacterial, antidepressant, anti-Parkinson's, anticancer, and anti-HIV activity.^91,92^ The usage of 2-oxazolidinones as chemical precursors in organic synthesis is also very common. Consequently, the synthesis of such therapeutic heterocyclic compounds has received a lot of attention.

Arshadi *et al.* synthesized toloxatone with the brand name Humoryl, 33 is an antidepressant drug that is marketed in several different countries comprising 2-oxazolidinones moieties as a structural unit. It functions as a reversible selective MAO-A (RIMA) inhibitor.^93^ These valuable heterocyclic composites are also widely used in the construction of organic compounds.^94^

**Scheme 15:**
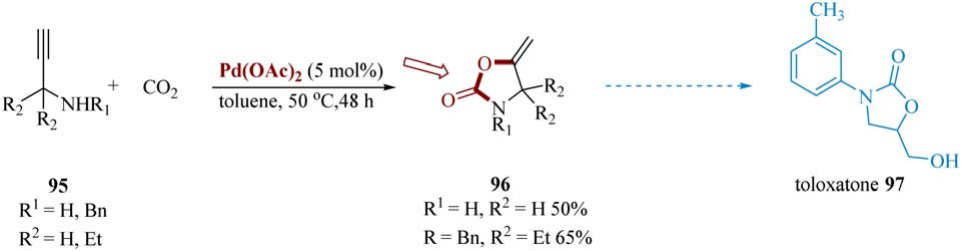
Synthesis of 5-(hydroxylmethyl)-3-(*m*-tolyl)oxazolidine 2-one (toloxatone).

Palladium-catalyzed carboxylation of secondary α,α-disubstituted *N*-propargyl amines 95 with CO_2_ produced the highly substituted 2-oxazolidinones 96. Pd(OAc)_2_, finest effective for the conversion, including Pd_2_(dba)_3_, Pd(PPh_3_)_4_, NiBr_2_(PPh_3_)_2_, IrCl(CO) (PPh_3_)_2_ and RuH_4_(PPh_3_)_2_. Substituted oxazolidinones 96 were then converted to toloxatone 97 over subsequent steps (Scheme 15).^95^

Among the most effective tricyclic antidepressants for blocking serotonin and norepinephrine reuptake is clomipramine. Although it raises the risk of seizures at high dosages, it has been proven to be useful in curing obsessive-compulsive disorder.

**Scheme 16:**
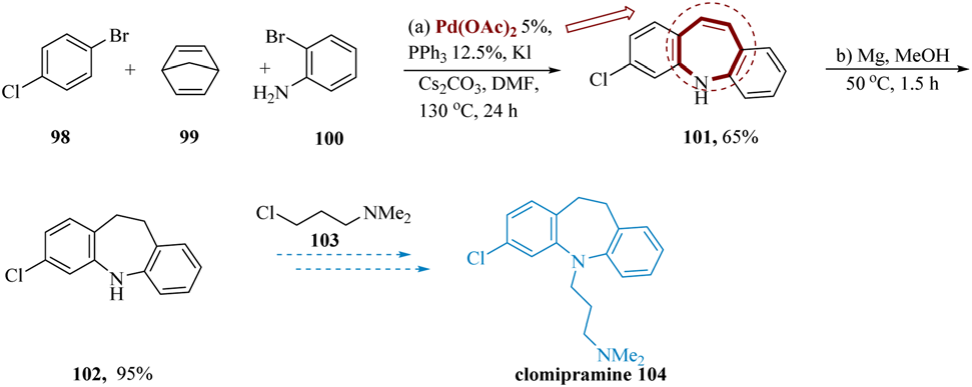
Synthesis of 3-(3-chloro-10,11-dihydro 5*H*-dibenzoazepin 5-yl)-*N*,*N*-dimethylpropane amine (clomipramine).

Because of their remarkable biological activity, 5*H*-dibenzo[*b*,*f*]azepines are the primary pharmacophore in clomipramine.^96–98^ Casnati *et al.* reported the formal synthesis of clomipramine used commercially accessible 4-bromochlorobenzene 98, 2-bromoaniline 100, also norbornadiene 99, along with cesium carbonate and dimethyl formamide, to produce the anticipated 3-chloro-5*H*-dibenzo[*b*,*f*]azepine 101 in yield of 65%. This compound was easily transformed to the resultant dihydro compound 102 in 95% yield by a simple reduction of a conjugated (=)-bond using the multipurpose Mg in MeOH at 50 °C for 1.5 hours. Finally, 3-chloro-*N*,*N*-dimethylpropan-1-amine 103 can be employed in alkylation to obtain the desired Clomipramine 104 (Scheme 16).^99–101^

Lumateperone, also known as ITI-007, is a strong 5-HT2A antagonist, post-synaptic D2 antagonist, and inhibitor of serotonin transport that was formed *via* Intra-Cellular Therapies.^102^ In 2017, the US FDA gave global approval for the single-dose oral administration of schizophrenia treatment in adults. The 5-HT2A, D2, D1/GluN2B, and SERT receptors exhibit a significant selectivity for the tetracyclic quinoxaline-type substance.^103^

**Scheme 17:**
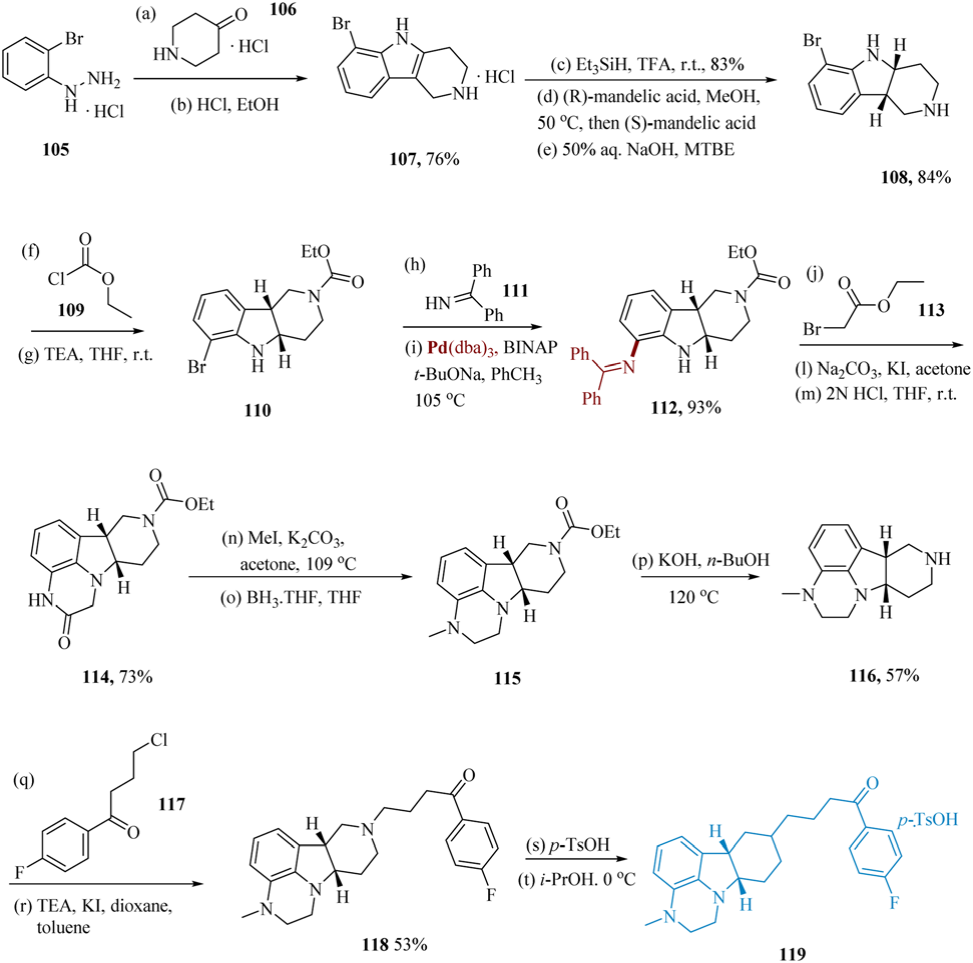
Synthesis of 1-(4-fluoro-phenyl)-4-((6*b*,10*a*)-3-methyl-2,3,6*b*,7,8,9,10,10*a*-octa-hydro-1*H*-pyrazino[3,2,1]carbazol-8-yl)butane-1-one (lumateperone).

Flick *et al.* described a simple and scalable pathway to lumateperone 119 and its structurally related compounds.^104^ Tricyclic indole 107 was produced using a Fischer indole with ketone 106 starting with hydrazine 105.^105^ Reduction with tri-ethylsilyl hydride, treating the resultant product with (*R*)-mandelic acid in MeOH, consequent synthesis of the (*S*)-mandelic acid diastereomeric salt, and free-basing with aq. NaOH yielded pure *cis*-indoline chirally 108. Protection of the amine and consequent Buchwald–Hartwig^106,107^ with 111 gave cyclization precursor 112. Tetracycle 114 was produced by spontaneous ring closure as a result of the *N*-alkylation of ethyl bromoacetate 113, following hydrolysis of the diphenylamine. Piperazine 115 was produced by again *N*-alkylation, following carbonyl reduction with borane in THF. The fully elaborated *cis*-tetracycle 118 was synthesized *via* hydrolysis of carbamate and *N*-alkylation using 4-chloro-1-(4-fluorophenyl)butane-1-one 117. Lumateperone tosylate 119 was produced by dissolving 118 in isopropanol and treating it with a *p*-toluene sulfonic acid solution (Scheme 17).^108,109^

**Scheme 18:**
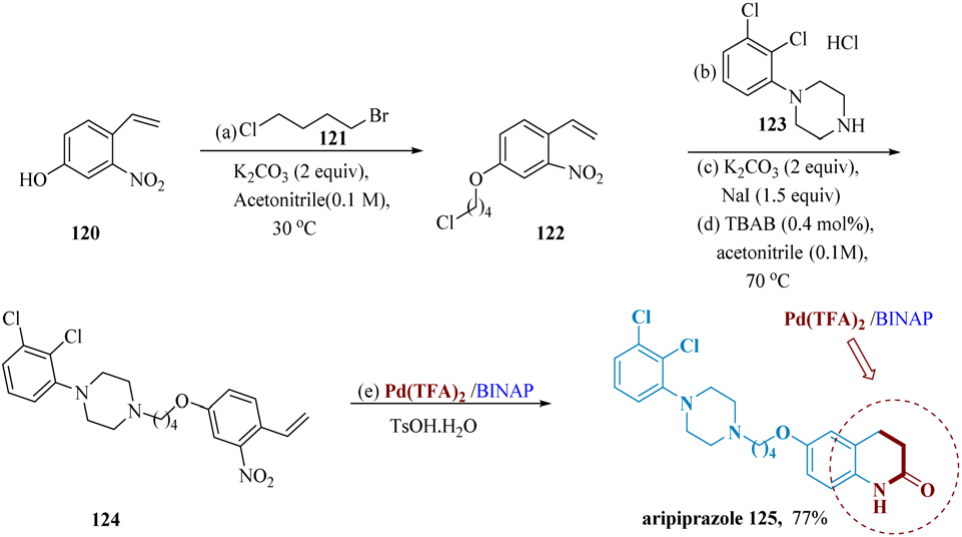
Synthesis of 7-(4-(4(2,3-dichloro-phenyl)piperazin-1-yl)-butoxy)3,4-dihydro quinolone 2(1*H*)-one (aripiprazole).

A new class of antipsychotic medication called aripiprazole (Abilify) is primarily utilized to cure schizophrenia and bipolar disorder.^110^ Because of the generation of difficult-to-remove isomers^111^ and the need for much more explosive Na azide as a nitrogen source and erosive TFA as the solvent, its conventional synthetic procedures usually gave poor yields. Yang *et al.* developed a faster synthetic pathway and milder reaction conditions allowed for a 77% total yield of aripiprazole using 120 as the initial substrate & current technology as the primary catalytic procedure. When the reaction was carried out in a Pd(TFA)_2_/BINAP/TsOH/H_2_O system, the NO_2_ group was completely deoxygenated and carbonylated to generate the isocyanate, which was then internally hydro-cyclized to produce aripiprazole 125 (Scheme 18).^110^

Milnacipran is a serotonin noradrenaline reuptake inhibitor (SNRI), and its amine-containing cyclopropane moieties display a variety of biological activities.^112–119^

**Scheme 19:**
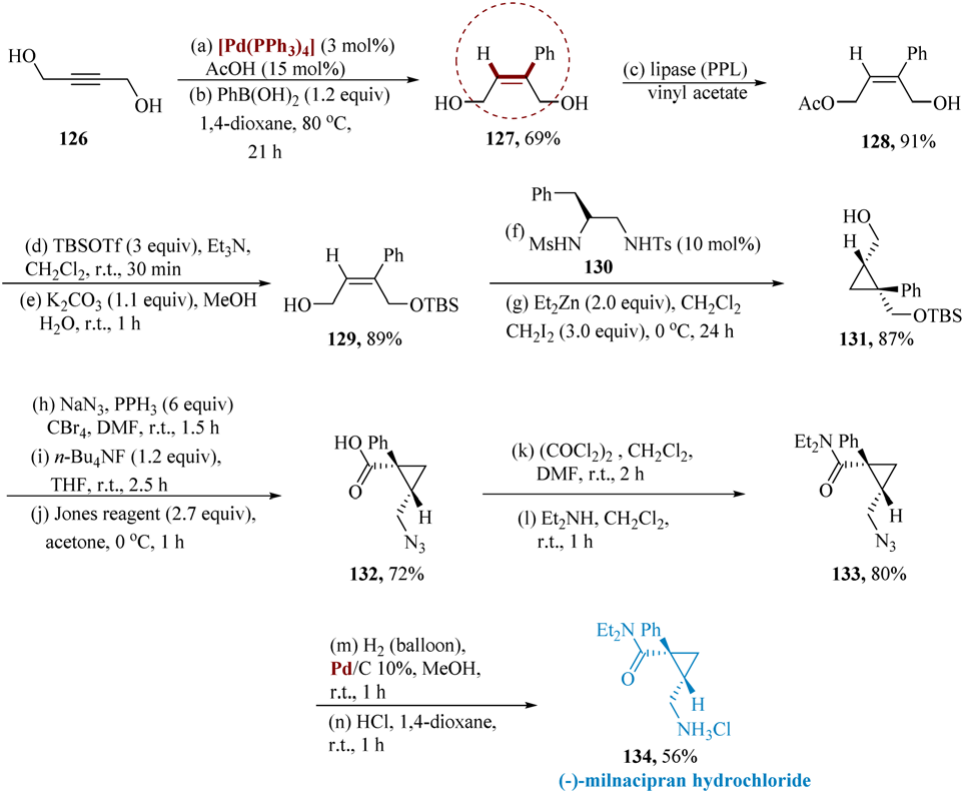
Synthesis of 2-((chloro-azanyl)methyl)-*N*,*N*-diethyl-1-phenyl cyclopropane carboxamide (milnacipran).

Ishizuka *et al.* reported an asymmetric production of milnacipran, initially, compound 129 was generated from radially available but2-yne-1,4-diol 126. By reacting with phenylboronic acid, compound 126 was transformed into (*Z*)-2-phenyl but-2-ene-1,4-diol 127. Using monoacetylation catalyzed by porcine pancreas lipase (PPL), the C4-hydroxy groups of 127 were regio-selectively preserved to provide 128. The C1-hydroxy group of 128 was protected with a *t*-butyldimethylsilyl group, and the C4-acetoxy group was then alkaline hydrolyzed to generate 129, which were then reacted with diiodomethane along with diethylzinc in 10 mol% of 130 to generate 131 with a yield of 87% (59% ee). Further primary hydroxy group of 131 was transformed into an azide; a *t*-butyldimethylsilyl group was then removed utilizing fluoride ions, and eventually, primary alcohol was oxidized utilizing Jones reagent to produce carboxylic acid 132. The carboxy group of 132 was transformed to amide 133, the azide group of 133 was hydrogenated, and the reaction with HCl provided the required optically active (−)-milnacipran hydrochloride 134 with 72% enantiomeric excess (Scheme 19).^120^

**Scheme 20:**
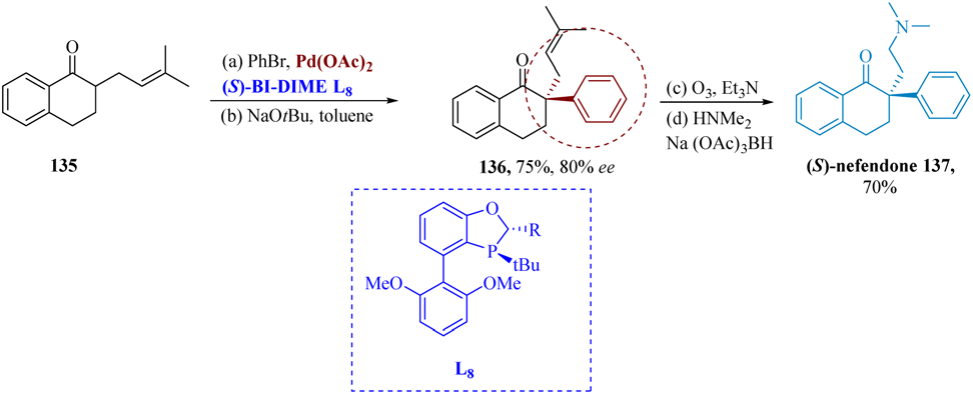
Synthesis of 2-(2-(dimethylamino) ethyl)-2-phenyl-3,4-dihydro naphthalen-1(2*H*)-one (nafenodone).

The absorption of norepinephrine and serotonin is potently and specifically inhibited by the drug nafenodone. Rao *et al.* described the effective synthesis of an antidepressant (*S*)-nafenodone. It was made possible by sterically hindered enantio-selective α-arylation *via* palladium driven by chiral mono-phosphorus ligand BI-DIME L_13_. Pd-(*S*)-BI-DIME serving as a catalyst, tetralone 135 reacted with PhBr to produce 136 in an 80% efficient and 75% yield. (*S*)-nafenodone 137 was produced *via* ozonolysis, reductive amination, and subsequent reaction with HNMe_2_/Na(OAc)_3_BH (Scheme 20).^121^

The universal α,β-esters were well-known as important olefin frameworks, and they were successfully used for parallel drug formation of (*Z*)-zimelidine. Ashida *et al.* reported the synthesis of an extremely selective serotonin reuptake inhibitor zimelidine, from readily available (*Z*)-stereo defined enol tosylates obtained from β-ketoesters 138 as well as α-formyl esters.^122^

**Scheme 21:**
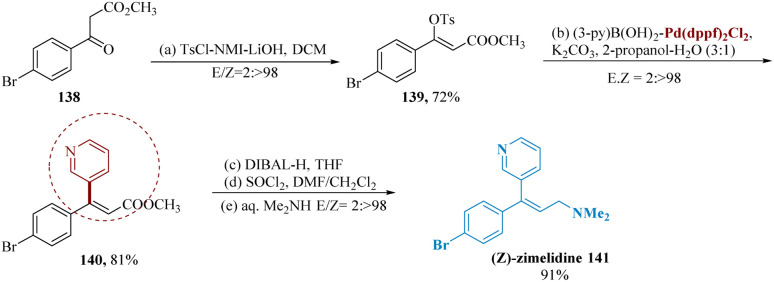
Synthesis of 3-(4-bromophenyl)-*N*,*N*-dimethyl-3-(pyridin-3-yl)prop-2-en amine (zimelidine).

Parallel and stereo-complementary enol tosylations were achieved *via* treating with starting material, undergoing Suzuki-couplings utilizing (3-Py)B(OH)_2_ which gave the compound 140. Our desired therapeutic molecule, (*Z*)-zimelidines 141, was then produced after being further treated with DIBAL-H, SOCl_2_, and aq. dimethyl amine (Scheme 21).^123^

3, 4-dihydro 2(1*H*)-quinolinones were marketed as an antipsychotic drug and exhibit promising antidepressant properties that are similar to those of aripiprazole.^124–127^ Triazole serves as the primary structural motif in a wide range of medicinal molecules, revealed to possess a variety of biological activities.^128,129^

In the FST and TST, the novel compound shows higher antidepressant efficacy than fluoxetine, as well as modest anticonvulsant action. These compounds could be employed as supplements to existing antidepressants to cure depression in epilepsy patients.

**Scheme 22:**
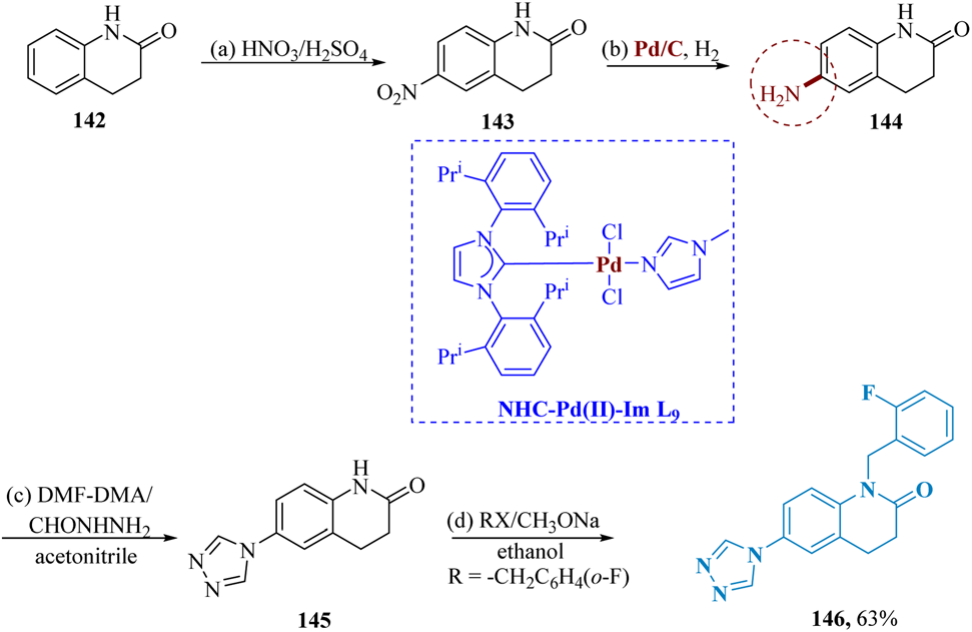
Synthesis of 1-(2-fluoro-benzyl)-6-(4*H*-1, 2,4-triazol-4-yl)-3,4-dihydro-quinolin-2(1*H*)-one.

Deng *et al.* described the synthesis of triazole-containing quinolinones. The readily accessible 3, 4-dihydro-2(1*H*)-quinolinone 142 underwent successive nitration and catalytic hydrogenation to synthesize the compound 144. Then, compound 144 was reacted with dimethoxy-*N*,*N*-dimethylmethanamine (DMF-DMA) as well as formyl-hydrazine in acetonitrile to produce compound 145. In contrast to fluoxetine, the target compound 146 was produced by the successive alkylation 145 with a range of diverse alkylating agents (Scheme 22).^130^

**Scheme 23:**
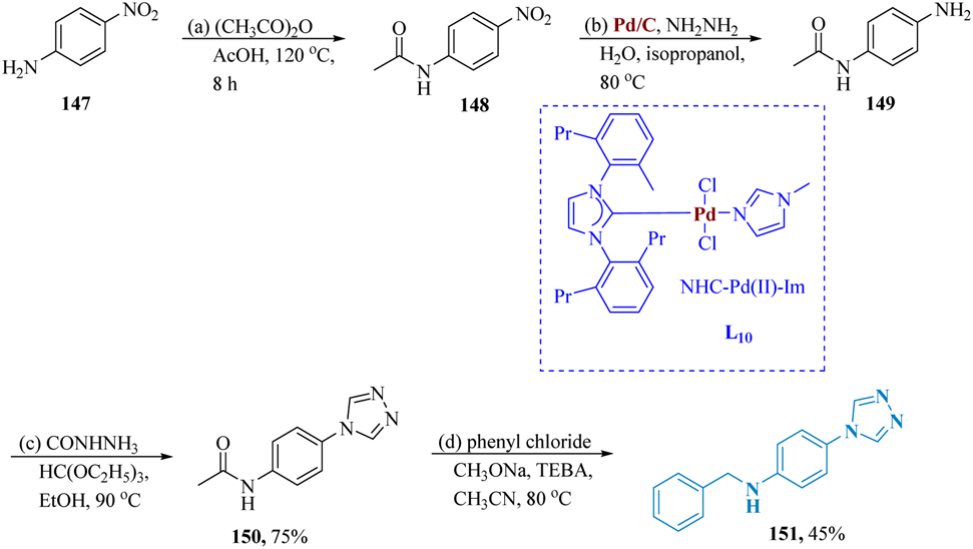
Synthesis of *N*-benzyl-4-(4*H*-1,2,4-triazol-4-yl)aniline.

Song *et al.* described the synthesis of triazole containing quinolinones as a potent antidepressant. Starting with nitroaniline 147 and treating it with propionic anhydride & acetic anhydride in refluxed acetic acid to produce the compound 148. NO_2_ reducing conditions of Pd/C as well as hydrazine hydrates were used to reduce compound 148 to produce compound 149. After that, compound 149 was treated with formyl hydrazine and triethyl orthoformate in acetonitrile to produce compound 150. Finally, compound 151, which was produced by alkylating compound 150 with a range of diverse alkylating agents, demonstrated greater antidepressant ability than fluoxetine in the TST and FST studies (Scheme 23).^131^

Clinical research on psychiatric disorders and migraines has concentrated on NK1 receptor antagonists.^132^ SSRIs are currently employed to cure mental illnesses because they prevent the uptake of 5-HT, which increases levels of 5-HT inside synaptic cleft.^133,134^ A novel category of antidepressants with therapeutic potential may be produced by combining SR inhibition mostly with modification of 5HT activity through NK1 antagonist.^135–137^

Risatti *et al.* reported the optimal α-arylation process which involves the formation of lithium enolate 153 using lithium dicyclohexyl amide accompanied *via* palladium with 154 utilizing tri-*tert*-butyl phosphonium tetra-fluoroborate as a ligand to provide ester 155, That was then reduced using lithium aluminum hydride to generate alcohol 156 in yields of 77%. LAH was also used to convert *N*-Boc to *N*-methyl, with a yield of 78–82%.

**Scheme 24:**
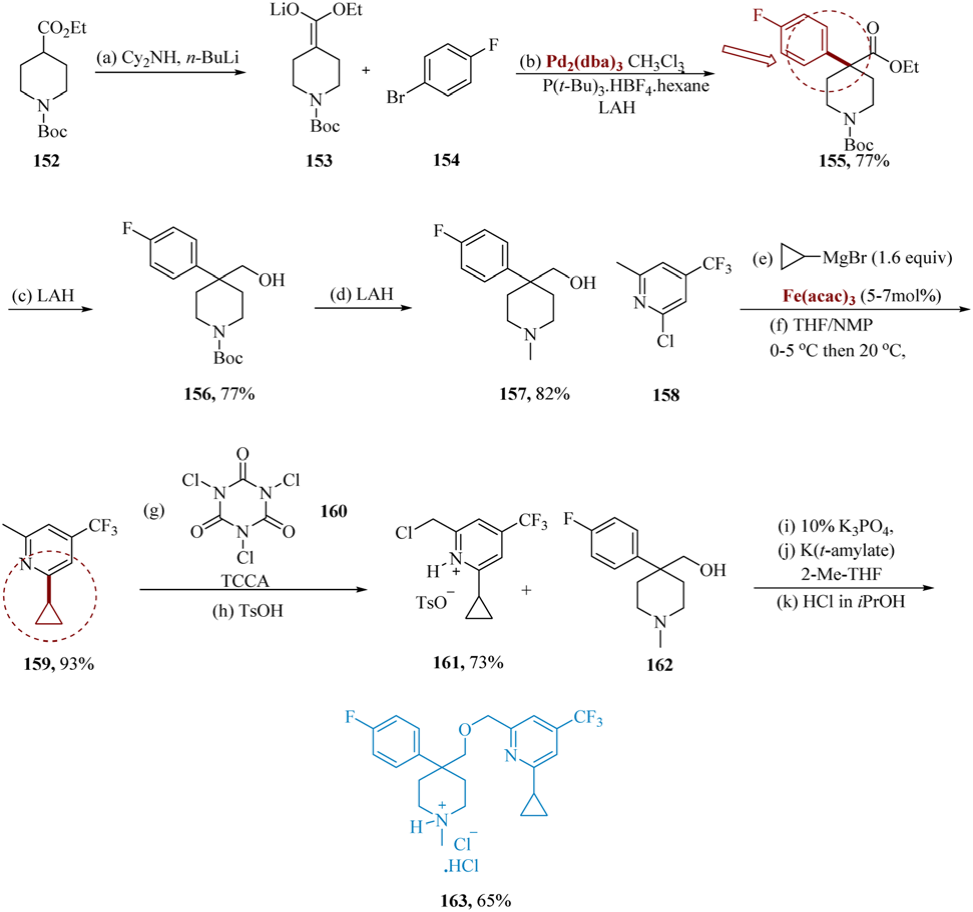
Synthesis of 4-(((6-cyclo-propyl-4-(trifluoro-methyl)-pyridine-2-yl)methoxy)methyl)4-(4-fluoro phenyl)-1-methyl piperidin-1-ium chloride (1 HCl) dual NK-1/serotonin receptor antagonist.

Through an iron-catalyzed coupling and TCCA chlorination, the 2,4,6-trisubstituted pyridine is synthesized selectively. However, after being treated with trichlorocyanuric acid 160, pyridine 159 was transformed into the necessary benzyl-chloride 161. Following the reaction, benzyl chloride 161 was produced as its *p*-toluene sulfonic acid (*p*-TSA) salt, including a dichloro impurity (5% LCAP) and leftover starting molecule 159. Finally, salt of *p*-toluenesulfonic acid 161 was synthesized from 158 with a yield of 68%, requiring just two synthetic transformations as opposed to the Boekelheide method's four stages, which resulted in a yield of 54%. Additionally, the method for coupling completely functionalized pyridine and piperidine components was extremely convergent, evaded the processing of non-crystalline products, and needed no chromatographic purifications. The crystalline HCl salt of 163, that were separated in 61–65% yield, was produced by etherifying the potassium alkoxide of 157 with a free base 162 (Scheme 24).^138^

## 5. Gold-catalyzed reactions

**Scheme 25:**
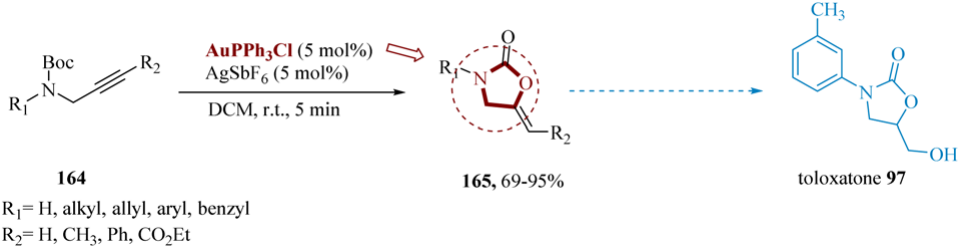
Alternative synthesis of toloxatone.

Vessally *et al.* reported a metal-catalyzed intra-molecular cyclization of *N*-Boc-protected propargyl amines using the AuPPh_3_Cl/AgSbF_6_ combination as the catalytic system to produce functionalized 2-oxazolidinones.^139^ Other catalysts, such as Pt(CH_3_CN)_2_(SbF_6_)_2_ and AuCl_3_, were discovered to increase the reaction in the optimization study, however, Au(PPh_3_)SbF_6_ provided the best results. *N*-Boc-protected propargyl amines 164 produced alkylidene 2-oxazolidinones 165 with fair to high yields and exceptional (*Z*)-selectivity under optimal conditions. Other merits of this synthetic methodology included simplicity, low reaction times, and a wide range of substrate scope. For instance, in the production of the antidepressant toloxatone 97 (Scheme 25).^140^

## 6. Manganese-catalyzed reactions

Desipramine is employed to treat depression, that works by enhancing the activity of a chemical called norepinephrine in the brain. This medication is a tricyclic anti-depressant. It might also be suitable to cure indications of attention-deficit hyperactivity disorder (ADHD).^141^

**Scheme 26:**
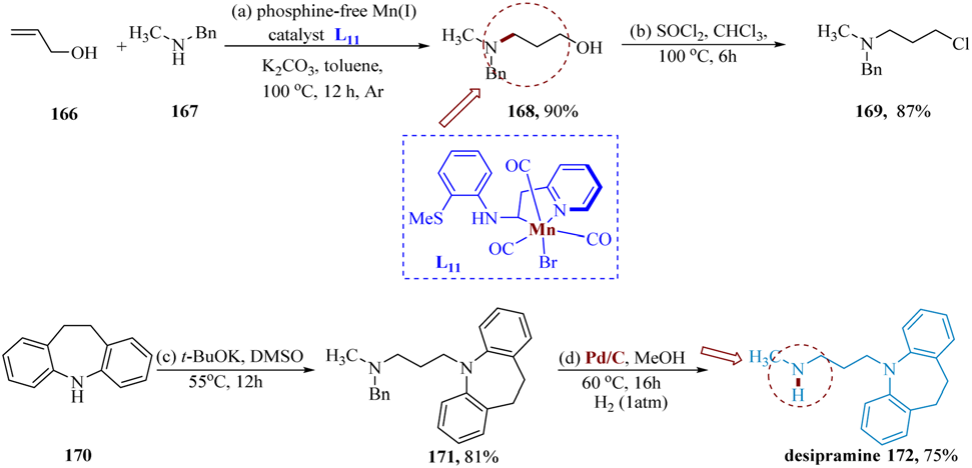
Synthesis of 3-(10,11-di-hydro 5*H*-dibenzo azepin 5-yl)*N*-methyl propane amine (desipramine).

Das *et al.* synthesized the precursor molecule 168 with exclusive anti-Markovnikov selectivity produced by hydrogenating allyl alcohol 166 with *N*-methylated aniline 167 and was transformed to chloro derivative 169 in yield of 87%, which was catalyzed *via* phosphine free Mn(i) L_11_ complex found abundantly in Earth and was carried out under hydrogen-borrowing conditions. Then, imino-dibenzyl treatment and debenzylation produced the antidepressant medication desipramine 172 in two steps with combined yields of 61% (Scheme 26).^142^

**Scheme 27:**
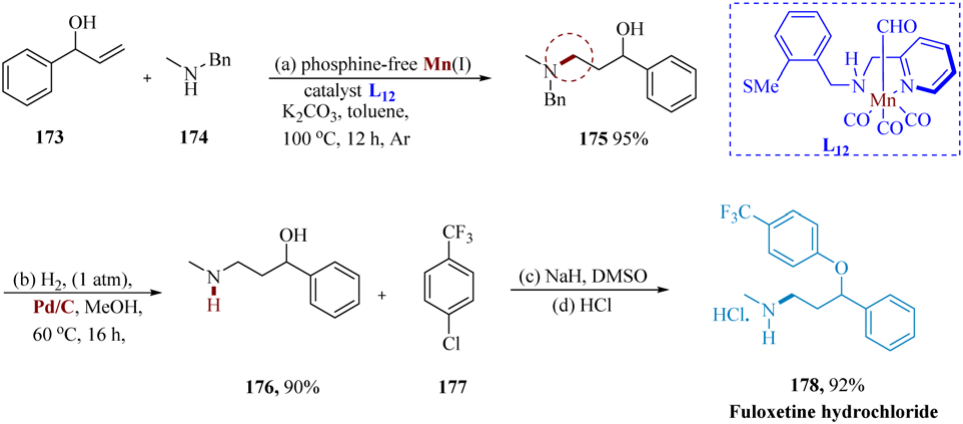
Synthesis of *N*-methyl 3-phenyl-3-(4-(trifluoro-methyl)phenoxy)propane amine (fluoxetine).

γ-Amino alcohols serve as efficient synthetic intermediates for a variety of drugs and bioactive compounds.^143^ Das *et al.* reported the synthesis of fluoxetine using a phosphine-free Earth's abundant Mn(i) catalyst. Under hydrogen-borrowing conditions, Mn(i) composite catalyzed the selective hydro-amination of allyl alcohols & 2° allylic alcohols with exceptional functional compatibility. 3-Benzyl(methyl)amino phenyl propane-1-ol 175, produced by treating 1-phenylprop-2-en-1-ol 173 with *N*-methyl-1-phenylmethanamine 174 and subjecting it to Mn, is then hydrogenated by using Pd/C in methanol at 60 °C for 16 hours. When the amine 176 was treated with 4-chlorobenzotrifluoride 177, it produced 178, as the hydrochloride salt (Scheme 27).^142^

Vortioxetine is a member of the bis-aryl-sulfanyl amines class and is chemically known as 1-[2-(2,4-dimethyl-phenylsulfanyl)-phenyl]-piperazine. Its main effects are the direct modulation of the 5-HT receptor and the selective blockade of SR (by inhibiting the SERT).^144^

**Scheme 28:**
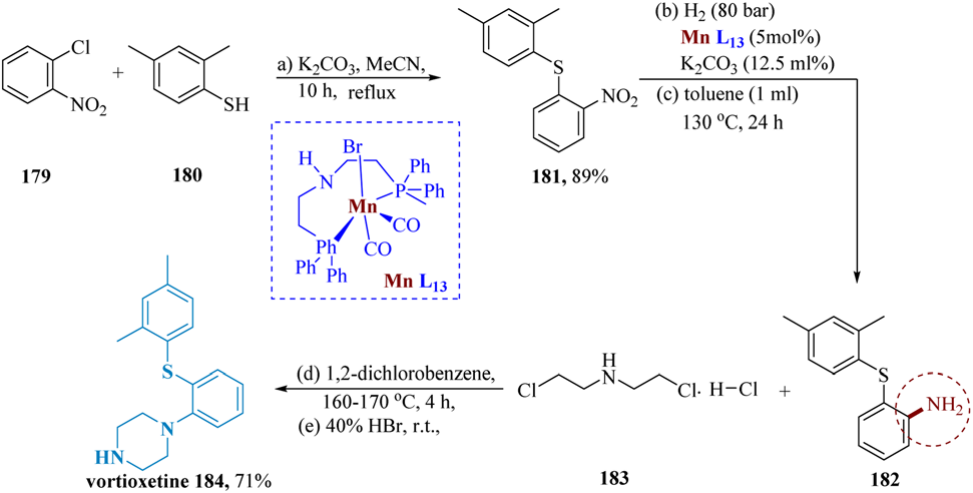
Synthesis of 1-(2-((2,4-dimethyl phenyl) thio)phenyl)piperazine (vortioxetine).

Mao *et al.* reported the synthesis of vortioxetine hydrobromide on a hectogram scale. Starting with 2,4-dimethylbenzenethiol 180 and 1-chloro-2-nitrobenzene 179, both of which are readily available in the market, the reaction with potassium carbonate in acetonitrile produced the desired intermediate 181 that was then purified through recrystallization in acetonitrile to yield the pure product in a total yield of 89%.^145^ The required the aniline derivative 182 is produced in 74% yield when the generated nitrophenylsulfane derivative 181 undergoes catalytic hydrogenation in the presence of Mn-1 under the optimal reaction conditions. It is worth noting that the activity of transition metals is frequently impeded by thio- and amino groups. Additionally, several Mn(0) species produced in late transition-metal catalyzation processes experience (C–S) oxidative additions which can be avoided by using Mn-L_13_. The required vortioxetine 184 is then produced by the reaction of 182 with 2-chloro-*N*-(2-chloro-ethyl)ethane amine hydrochloride 183 (Scheme 28).^146,147^

## 7. Copper-catalysed reactions

**Scheme 29:**
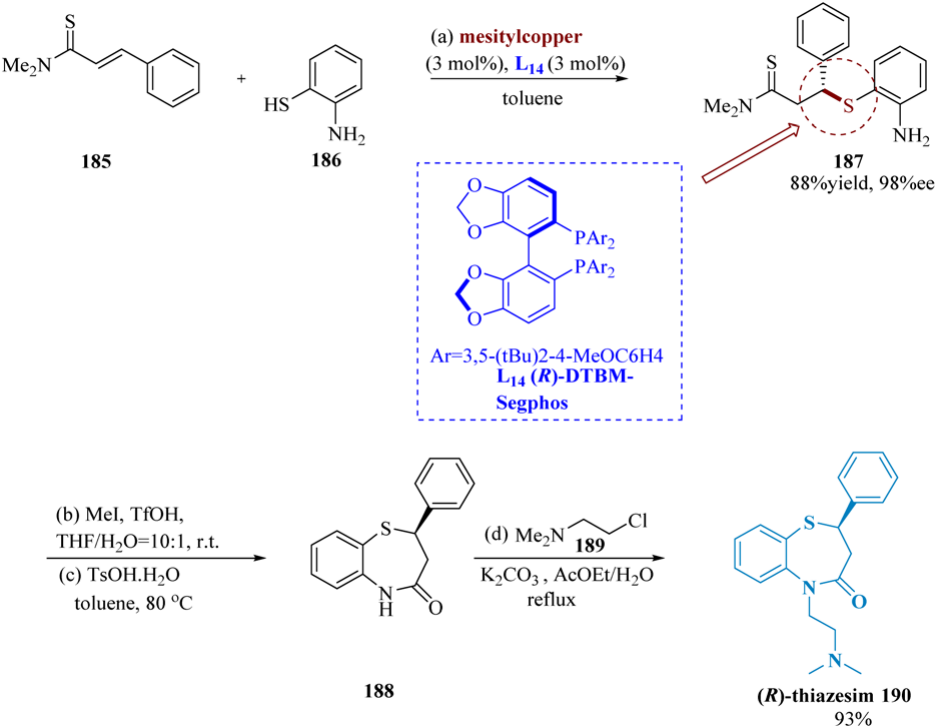
Synthesis of 5-(2-(dimethyl-amino)ethyl)2-phenyl-2,3-di-hydro benzo[1,4]thiazepin-4(5*H*)-one (thiazesim).

In many biological molecules, 1,5-benzo thiazepines are preferred heterocyclic pharmacophores.^148^ Ogawa *et al.* described speedy accessibility to 1,5-benzothiazepines using mesityl copper/(*R*)-DTBM segphos (DTBM = 3,5-di-*tert*-butyl-4 methoxy), pre-catalyst for conjugate addition of α,β-unsaturated thioamides 185 & thiophenol 186. The complex of mesitylcopper and (*R*)-DTBM segphos L_1_ may function as effective catalysts for direct enantio-selective production of C–S bonds. Several 1,4-conjugate addition compounds were produced by successfully using a range of electron-rich and deficient α,β-unsaturated thio-amides as electrophilic substrates in toluene at 0 °C. The second conversion required the utilization of methyl iodide for methylation of thio-amide functionalities, which produced a transitory thioester that was cyclized at 80 °C using a catalytic proportion of *p*-toluene sulfonic acid monohydrate (TsOHH_2_O). Following side chain addition, a 93% yield of (*R*)-thiazesim 190 was achieved (Scheme 29).^149^

Reboxetine, a selective norepinephrine reuptake inhibitor1 (SNRI), is used to treat depression, narcolepsy, cocaine dependence disorder, and hyperactivity disorder.^150,151^ In contrast to its (*R*,*R*) enantiomer, (*S*,*S*)-reboxetine is much more potent and specific for both nor-epinephrine transporters.^152^

**Scheme 30:**
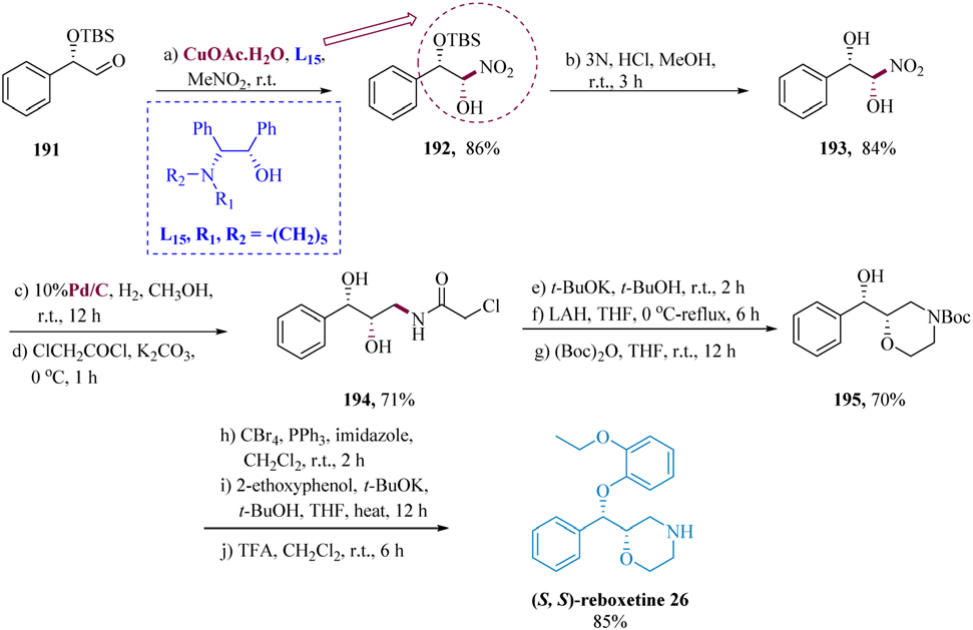
Synthesis of (*S*)-2-((*S*)-(2-ethoxy phenoxy)(phenyl)methyl)morpholine (reboxetine).

Liu *et al.* used Cu-L_6_ chiral amino alcohol-copper(ii) catalyst to facilitate the diastereoselective nitro-aldol reactions of nitromethane with chiral aldehyde, which potentially leads to the privileged synthesis of specific stereoisomer for nitro-diol derivatives, Cu-L_15_ chiral amino alcohol-copper(ii) catalyst was used. The nitro-aldol adduct (1*S*,2*S*) 192 was produced in 86% yield when the aldehyde (1*S*,2*S*) 191 was reacted with CH_3_NO_2_ in the presence of Cu-L_6_. The O-TBS-protected molecule 192 was first deprotected with 3 N HCl to generate the diol 193, which was then hydrogenated with Pd/C and subjected to a series of treatments with ClCH_2_COCl while being accompanied by a base to yield the chloroacetamide derivative 194 in 71% yield. The morpholine derivative (2*S*,3*S*) 195 was developed in 70% by cyclizing the amide derivative (2*S*,3*S*) 194 with *t*-BuOK, then reducing the amide with LAH and protecting it with *N*-Boc. Ultimately, derivative 195 was converted to (*S*,*S*) reboxetine 26 in 85% yield (Scheme 30).^153^

**Scheme 31:**
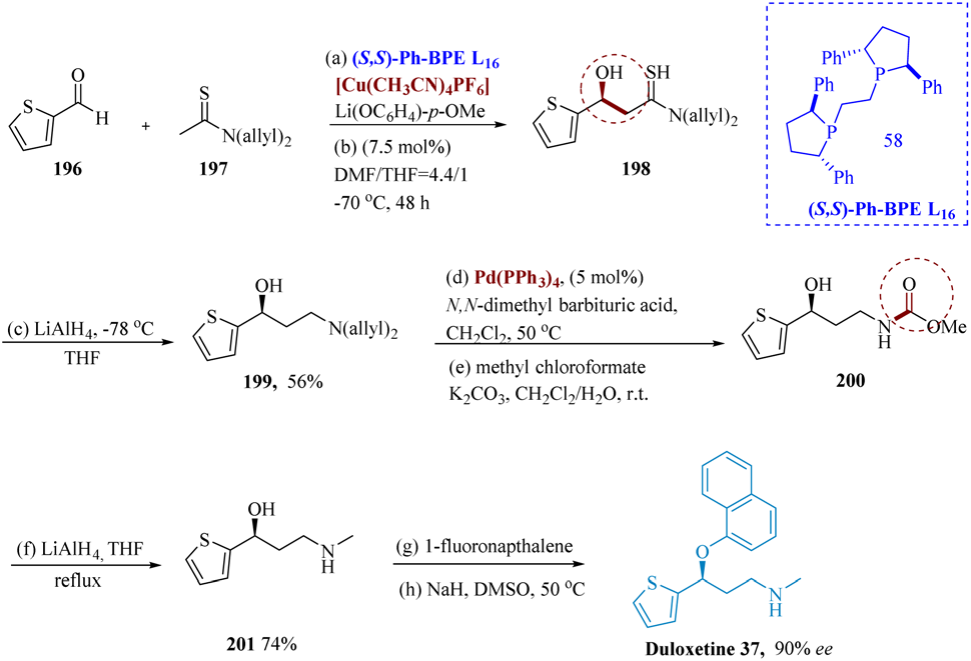
Synthesis of (duloxetine).

Duloxetine, a powerful antidepressant for the treatment of serious depressive disorders.^59^ Inhibitors of serotonin and nor-epinephrine reuptake for the treatment of several illnesses associated with depression.^154,155^ Larik *et al.* reported that thio-amides are employed as significant precursors for (C–C), leading to an aldol product having 92% ee for the enantio-selective direct asymmetric aldol reaction that produces (*S*)-duloxetine. The chirality has been produced by constructing a soft Lewis acid/hard Brønsted base co-ordinated catalyst consisting of [Cu(CH_3_CN)_4_]PF_6_, (*S*,*S*)-PhBPE L_16_, and Li(OC_6_H_4_-*p*-OMe), where thioamide was chemoselectively activated *via* soft–soft interaction of Cu^+^ and sulfur atom, resulting in the unique production of the thioamide enolate in aldehyde which undergoes reduction and synthesized compound 199. Molecule 200 was produced in two steps using 5 mol% of Pd(PPh_3_)_4_ and *N*,*N*-dimethyl of barbiturates in DCM at 50 °C. This molecule then underwent LiAlH_4_ reduction to produce molecule 201. The final target product 37 was then produced with a 65% yield by adding sodium hydride and 1-fluoro naphthalene (Scheme 31).^156^

Yang *et al.* reported the synthesis of triple reuptake inhibitor (TRI) ALB 109780, which prevents the reuptake of serotonin, norepinephrine, and dopamine, may help cure depression.^157^

In the proximity of potassium carbonates, copper(i) iodide as well as l-proline in DMS (dimethyl sulfoxide), the reaction between compounds 202 and 203 produced the intended product 204 as the light-yellow limpid solid in 68% yield. A preliminary analysis of the reaction situations for the α-arylation of 204 showed that BINAP, NaO*t*-Bu, and Pd(OAc)_2_ were the best catalysts for this reaction. Compared to toluene, dioxane often offered superior adaptation and crude purity. Regardless of the quantity of catalyst utilized, increasing the addition of compound 205 harms the reaction's crude purity. Higher base concentrations resulted in improved conversion. With 1.2 equiv. of compound 205 at 80 °C, 5% Pd(OAc)_2_, BINAP as the ligand in dioxane, and 1.5 equiv. of NaO*t*-Bu, the best results were obtained.

Borane-dimethylsulfide (BMS) was used to reduce 206 in the presence of THF and 6 N HCl at 35 °C in a nitrogen environment. The resultant solution was then stirred at 40 °C till it was finished. Over the reduction and treatment with charcoal, compound 207 was separated also as a yellow solid with a 66% yield. After the treatment, purity rose to 95.4% from 87.5%.

**Scheme 32:**
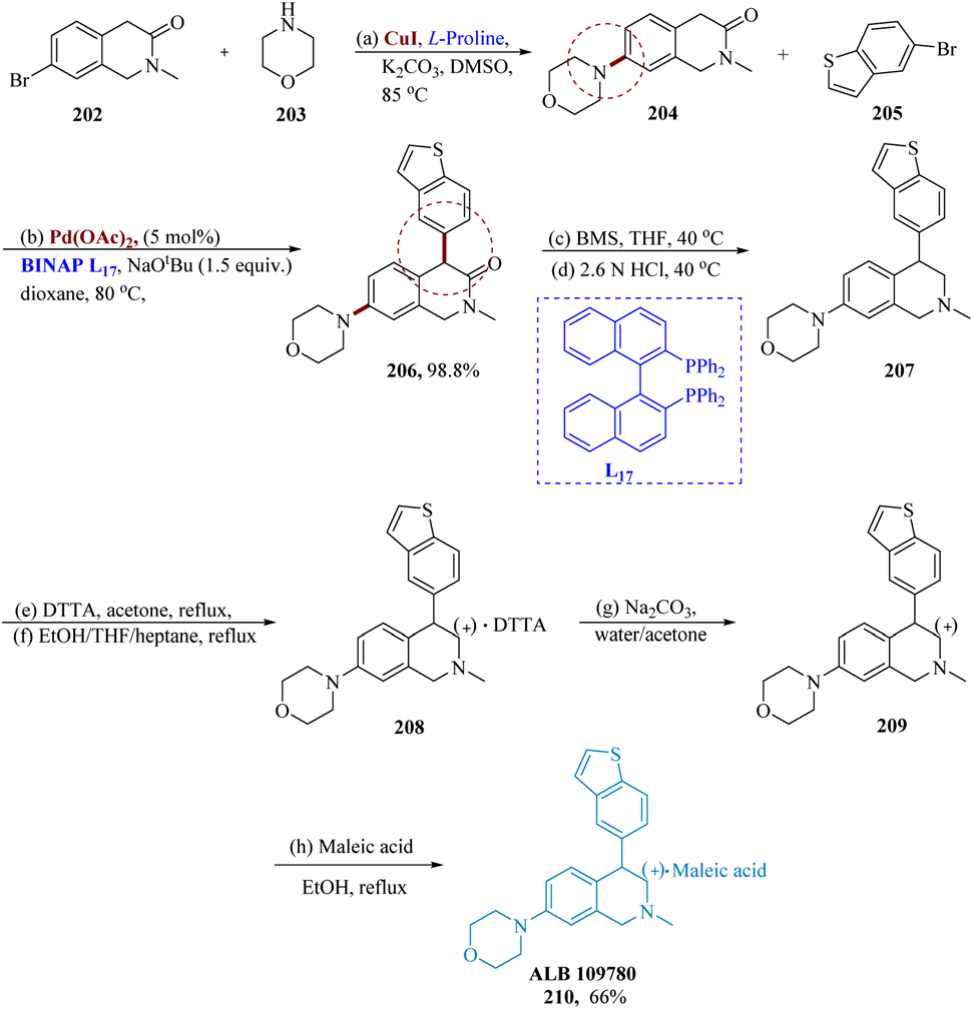
Synthesis of ALB 109780, a triple reuptake inhibitor.

By adding (+)-DTTA to a racemic 207 solution in acetone at reflux, 207 was resolved using (+)-di-*p*-toluoyl-d-tartaric acid. The resultant solution was chilled to 5 °C to yield the required product as the white solid *via* filtration in 81 to 88% ee. After the isolated product was recrystallized using heptane as an anti-solvent to speed up crystallization and 10% THF/EtOH at reflux (68 °C), the chiral limpidness was significantly improved to 98.8% enantiomeric excess. On a scale, 208 was reacted with 6 equiv. of Na_2_CO_3_ solution while being immersed in a mixture of aq. acetone. The resulting suspension underwent filtration to provide 97.1% HPLC purity and a quantitative yield of the target product. It was discovered that the chiral purity was 94.3%. The free base 209 was allowed to react with a solution of 1.1 equivalent to maleic acid after being heated to reflux in ethanol. It was filtered after cooling to 5 °C, yielding the anticipated product 210 as a white solid with a yield of 91% and HPLC purity of 98.1%. After isolation, the chiral purity was found to be greater than 99.9%. Additionally, small-scale investigations showed that during salt generation, from the starting substrate with only 86% ee, the chiral purity is sometimes increased to 99% (Scheme 32).^157^

Uwamori & Nakada synthesized Hyperforin, which was derived from *Hypericum perforatum* L., which prevents synapses from reabsorbing neurotransmitters.^158,159^ The compound known as hyperforin, which belongs to the family of poly-prenylated acyl phloroglucinols (PPAPs), is composed of a strongly oxygenated and highly substituted bi-cyclo[3.3.1]nonane or bicyclo[3.2.1]octane motif using geranyl or prenyl side chain derivatives prepared by intramolecular cyclopropanation.^160,161^

Intra-molecular cyclopropanation (IMCP) of 211, produced from methyl 2,6-dimethoxy benzoate by a series of steps, was accompanied by stereo-selective alkylation of the cyclopropane 212*via* copper catalyzed complexes with 2,2′-(propane-2,2-diyl)bis(4,5-dihydro oxazole), and region-selective ring opening of a cyclo propane moiety to afford 213. Compound 213 was transformed to compound 217 through chemo-selective as well as stereo-selective hydroboration of 213 using disiamyl borane for further protection of the subsequent hydroxyl as TIPS ether generated 214. Synthesis of an enol triflate of 214 using Comins' reagent and Pd carbonylation provided 215. By hydrogenating the C6–C7 alkene in 215 using Crabtree's catalyst, chemo- and stereo-selective reduction of the C6–C7 alkene were accomplished, leading to the only product 216 after refluxing dichloroethane. The directing effect of a C2–C3 methoxy alkene may be responsible for this stereoselectivity. Preferential acetylation of a 1° hydroxyl and then Dess–Martin oxidation of a C-9 secondary hydroxyl produced product 217 from the reduction of 216 with DIBAL-H. Palladium-catalyzed oxidation of 217 was used to achieve allylic oxidation, yielding 218, which was then modified by Dess–Martin oxidation, Wittig reaction, and elimination of the TBS group to yield 219. As mentioned earlier, Wittig reactions were also used to synthesize the allyl group at the C-7 position. In other words, potassium carbonate in methanol removed the acetyl groups from 219, which were then proceeded *via* DMP and Wittig process to give 220. After compound 220 underwent acid hydrolysis, compound 221 with an allyl group at the C-7 position was successfully produced by the Wittig reaction. Lithium 2,2,6,6-tetra-methylpiperidide was necessary for allylation at the C-5 position of 221, as LDA reduced the C9 ketone. Under the same circumstances, subsequent allylation at a C-3 site did not occur, requiring the utilization of thienyl cuprate as an additive, thus giving compound 222. Compound 222 was exposed to a reaction with TBAF to construct the C-1 isopropyl ketone, and a Dess–Martin oxidation process followed to produce aldehyde 223.

The required product was successfully obtained with a trace amount of the reduced product from the treatment of molecule 223 with the isopropyl cerium, which was successfully prepared *in situ* from CeCl_3_·2LiCl and isopropyl magnesium chloride. Cross metathesis was subjected to 224, which was produced by the subsequent Dess–Martin oxidation. In the Grubbs II reagent at 60 °C, the reaction of 224 with isobutene produced a variety of products, some of which comprised compounds with cycloheptene rings that were obtained from the ring-closing metathesis among the C-7 and C-8 substituents.

**Scheme 33:**
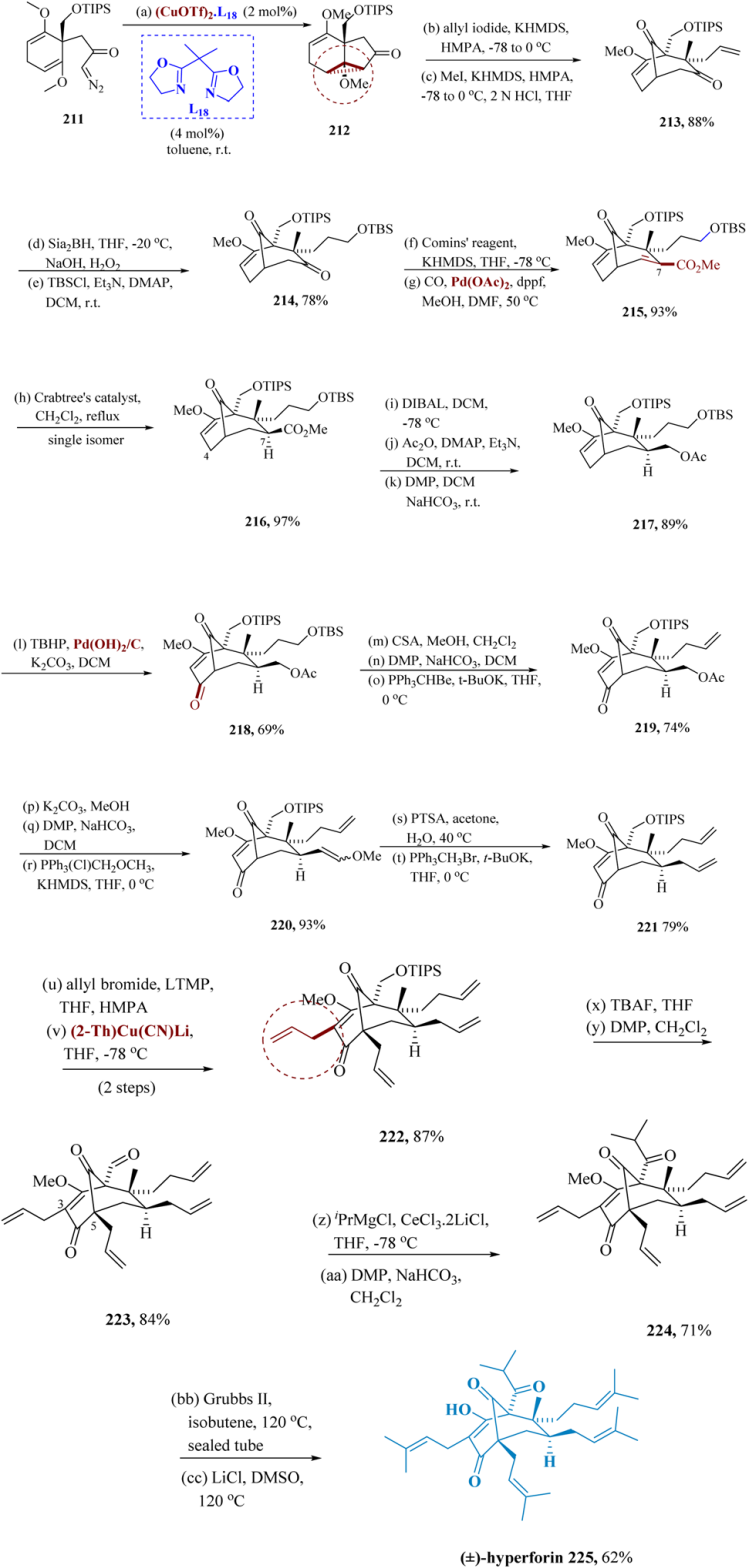
Synthesis of polycyclic poly-prenylated acylphloroglucinols (hyperforin).

Moreover, the target compound 225 was effectively produced in 93% yield at 120 °C. Under Krapcho's conditions, the C-2 methyl ether was finally cleaved (Scheme 33).^162^

## 8. Rhodium-catalysed reactions

**Scheme 34:**
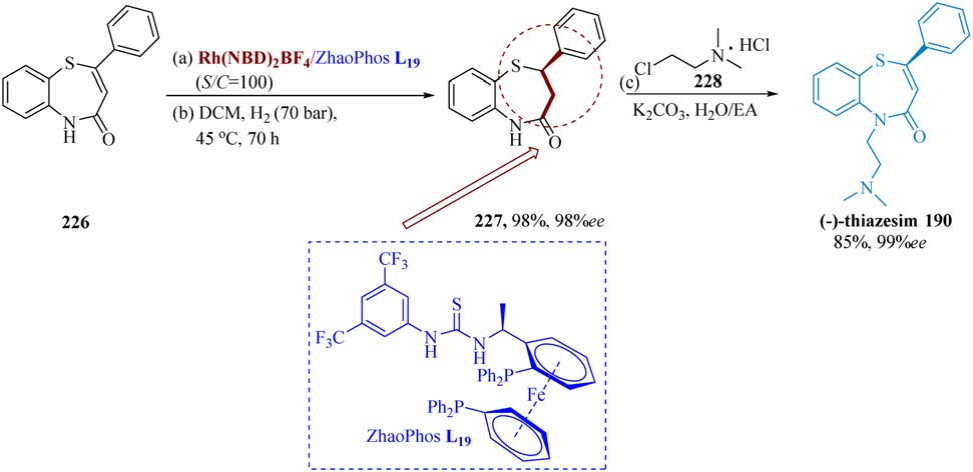
Synthesis of thiazesim.

A lot of attention has been attracted to the asymmetric synthesis of optically active 1,5-benzo thiazepines that have chiral drugs in the form of a sulfur-substituted stereocenter. Yin *et al.* successfully developed asymmetric hydrogenation of the number of conjugate C=C, using the Rh/ZhaoPhos catalytic system, which consists of the chiral ferrocene-based bis-phosphine & thiourea moiety as ligand. With an efficient S/C ratio of 100, asymmetric hydrogenation of 226 produced the compound (*R*)2-phenyl-2,3-dihydro benzo[*b*][1,4]thiazepin 4(5*H*)-one 227 having the yield of 98% (98% ee). The antidepressant drug (*R*)-(−)-thiazesim 190 could be easily converted from the hydrogenation product 227 with outstanding efficiency (Scheme 34).^163^

Venlafaxine, a member of the phenylethylamine class of antidepressants with a unique structure, has a chiral center at a benzylic position, a tertiary amine & a tertiary hydroxy group.^164,165^

**Scheme 35:**
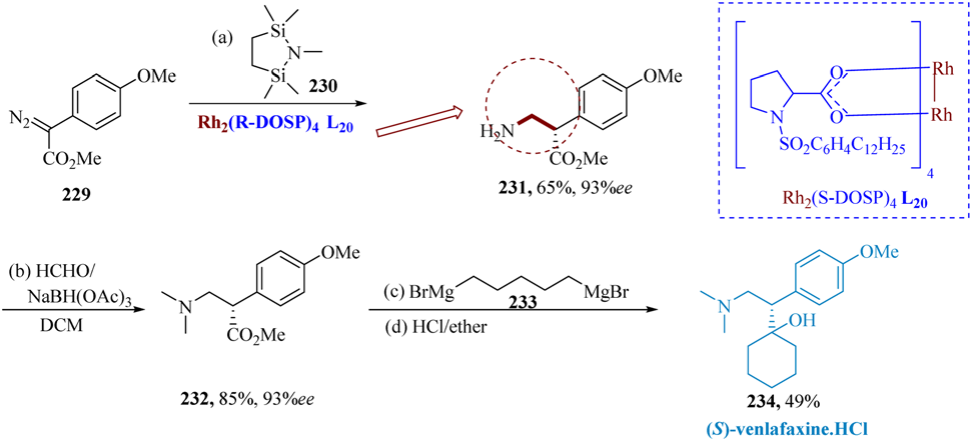
Synthesis of 1-(2-(dimethyl amino)-1-(4-methoxy phenyl)ethyl)cyclo-hexane-1-ol (venlafaxine).

Preskorn described The C–H insertion reactions of bis-silylmethylamine 230 using different aryl diazo-acetates 229 were catalyzed *via* dirhodium-(ii)-prolinate, Rh_2_(*S*-DOSP)_4_L_20_ and produced β-amino esters in 62% yield and 93% ee. It was observed that HCHO/NaBH(OAc)_3_ was a viable substitute since it effectively converted 231 to 232 in a yield of 82% at room temperature without losing ee. Finally, (*S*)-venlafaxine 234 was synthesized by reacting 232 with pentyl-1,5 magnesium bromide 233. The Grignard reagent and the ester solutions have to be added slowly and simultaneously to the reaction vessel to achieve the best results. After working up the reaction, producing the HCl salt, and enriching by recrystallization, (*S*)-234 was produced in a yield of 49% and 99% ee (Scheme 35).^166^

The antidepressant medicine escitalopram, also known as 3-(dimethyl amino)propyl-4-fluorophenyl-1,3-dihydro-isobenzofuran-5-carbonitrile, is one example of a pharmaceutical agent or natural product that contains chiral diaryl alkyl carbinol moieties.^167–172^ Huang *et al.* described for the first time addition of aryl-boroxines to modest aryl ketones in a highly enantioselective manner, using a Rh/(*R*,*R*,*R*,*R*)-WingPhos to produce chiral diaryl alkyl carbinols. It has been established that Rh/(*R*,*R*,*R*,*R*)-WingPhos is important for the significant reactivity and enantio-selectivity. Because of the two anthryl units in its composition, (*R*,*R*,*R*,*R*)-WingPhos can not only provide the necessary stereo-control but also help to bring two reactions together and increase reactivity.^173^

**Scheme 36:**
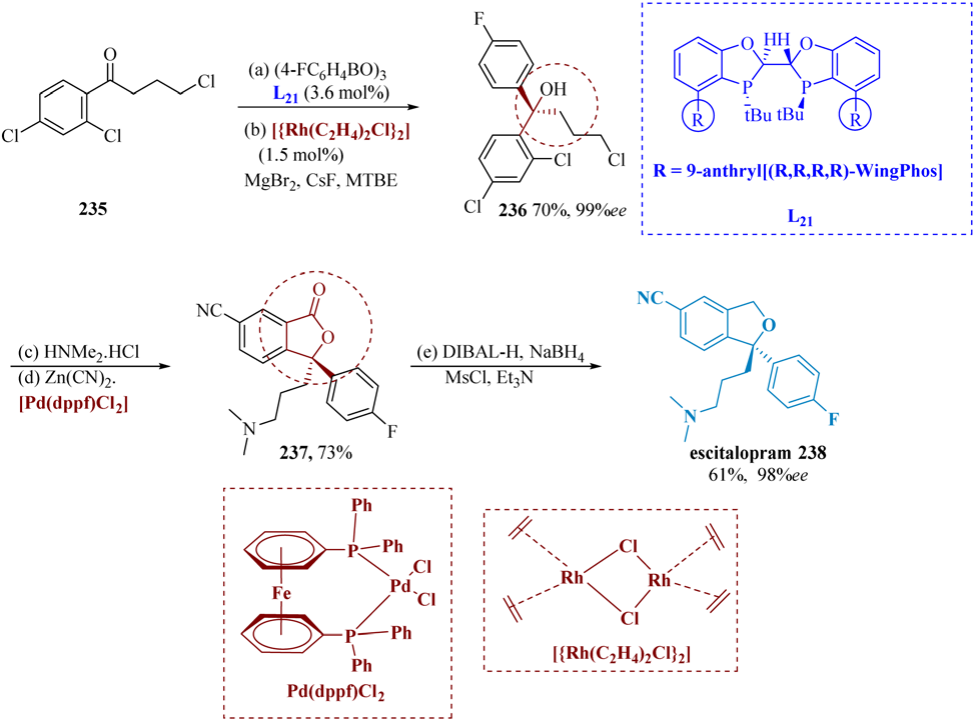
Synthesis of 1-(3-(dimethyl amino)propyl)-1-(4-fluoro phenyl)-1,3-dihydro-isobenzofuran-5-carbonitrile (escitalopram).

The targeted tertiary alcohol 236 was effectively produced in 70% yield (99% ee) by adding 4-fluoro-phenyl boroxine to 4-chloro-1-(2,4-dichloro phenyl)butan-1-one 235 using the Rh/(*R*,*R*,*R*,*R*)-WingPhos L_21_, which has excellent functional group compatibility. The lactone 237 was produced with a 73% overall yield by SN_2_ displacement of chloride in 236 with amines and then cyanation–lactonization under palladium catalysis. Escitalopram 238 was produced with a combined yield of 61% and enantiomeric excess of more than 98% after being reduced from 237 using DIBAL-H/NaBH_4_ and then having the ring closed with MsCl treatment. Thus, using this methodology, a fresh, brief, and extremely enantioselective synthesis of escitalopram was established (Scheme 36).

**Fig. 4:**
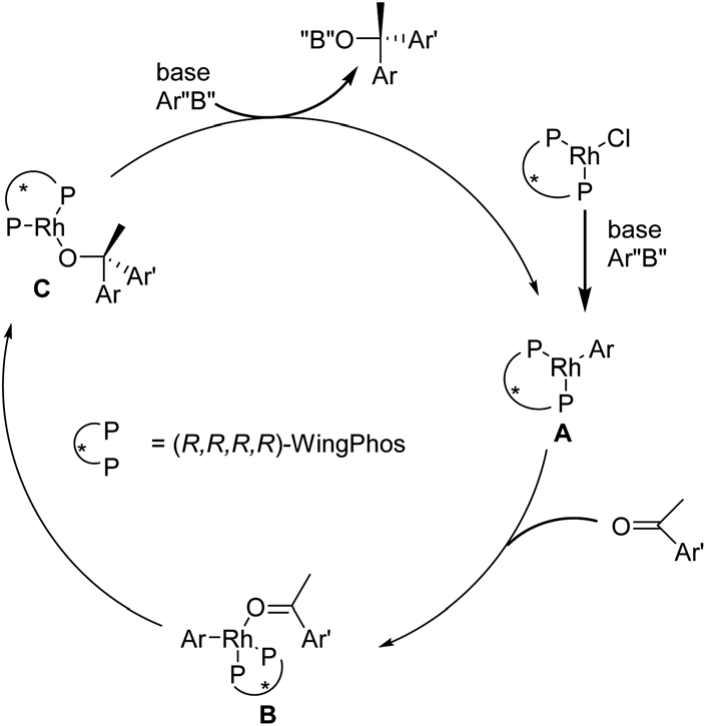
Mechanism for the synthesis of escitalopram.

A simplified mechanism for this rhodium-catalyzed asymmetric inclusion of arylboroxines to simple aryl ketones is proposed in Fig. 4. Transmetallation of the aryl boron with the [Rh(Cl){(*R*,*R*,*R*,*R*)-WingPhos}] species provides the aryl-Rh species A. This step is followed by the coordination of an aryl ketone to form the species B. The favorable conformer undergoes insertion and transmetallation with another aryl boron reagent to provide the chiral tertiary alcohol product with the ascertained stereochemistry and regenerates A (Fig. 4).^173^

**Scheme 37:**
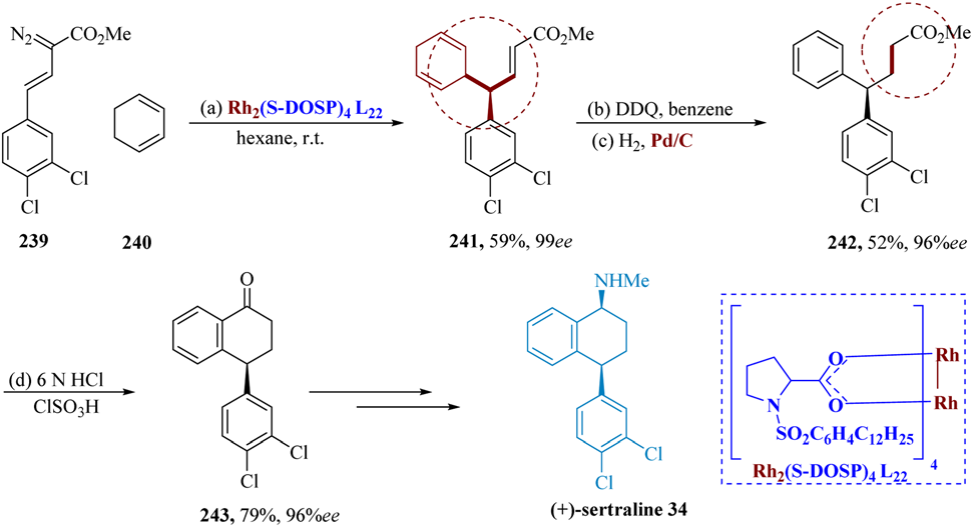
Synthesis of sertraline.

Davies *et al.* reported that cyclohexadienes can be successfully subjected intermolecularly to C–H insertion of phenyl diazoacetates *via* dirhodium tetrakis((*S*)-*N*-(dodecyl benzenesulfonyl)prolinate) (Rh_2_(*S*-DOSP)_4_) L_22_, resulting in the asymmetric production of diarylacetates. The 1,4-cyclohexadiene 240 was produced in 99% ee by the vinyl di-azoacetate 239 and 1,3-cyclohexadiene which was catalyzed *via* Rh_2_(S-DOSP)_4_L_22_. The use of cyclohexadiene 241 as a precursor for the formal synthesis of (+)-sertraline 34 has many advantages. The 4,4-diaryl butanoate 242 was produced by oxidizing 241 with DDQ and catalytic hydrogenation, with only minimal racemization (96% ee). The tetralone 243 was produced by ester hydrolysis of 242*via* an intra-molecular Friedel–Crafts acylation (79% yield for two processes), which was then transformed into (+)-sertraline 34 (Scheme 37).^174^

**Scheme 38:**
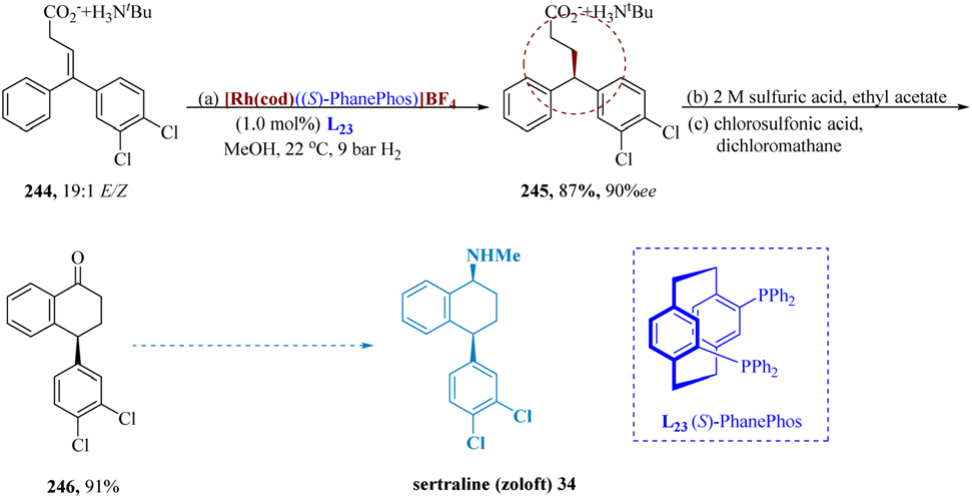
Another pathway for synthesis of sertraline.

Boulton *et al.* reported a potent method to construct stereo-genic centers is the asymmetric hydrogenation of the olefin functional group using rhodium di-phosphine catalysts and an (*S*)-PhanePhos ligand L_23_.^175^*Z*/*E*-olefin isomers produce different enantiomers in a given catalytic system, the compound 244 (19 : 1 *E*/*Z* mixture of olefins) needed to be employed as (almost) a single isomer to obtain excellent enantio-selectivity. The racemic form of 4,4-diaryl-3-butenoate 245 was produced by hydrogenating the tert-butylammonium salt 244 using a 1 : 1 *E*/*Z* mixture of olefins. The hydrogenated product was cyclized by first treating it with 2 M H_2_SO_4_ to release a free acid, and then with chloro-sulfonic acid to produce tetralone 246 91% of the time. Tetralone 246 was then employed to develop sertraline 34 by reductive amination with methylamine (Scheme 38).^176^

As a result of selective inhibition of the absorption of human synaptosomal serotonin, (+)-sertraline (Zoloft) has become the medication that is most usually used for the treatment of depression.^177,178^ It is frequently employed for treating depression, as well as occasionally OCD, PTSD, and panic attacks.

**Scheme 39:**
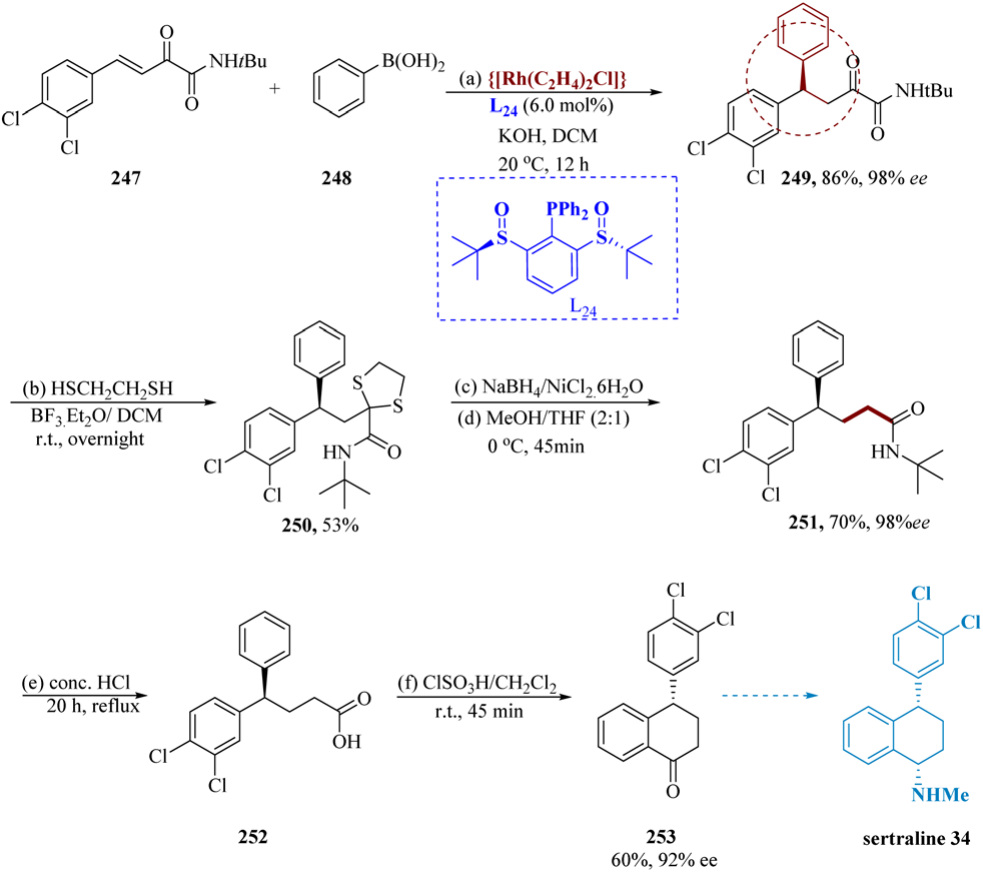
Synthesis of sertraline.

Wang *et al.* described the production of chiral gem-diaryl alkanes in significant yield and enantiomeric excess was enabled by chiral sulfinylphosphine ligands, which successfully promoted Rh-catalyzed arylation to chalcones. In aqueous KOH in dichloromethane (DCM), phenylboronic acid 248 has been introduced to the β,γ-unsaturated α-ketoamides & ligand L_24_. Rh(C_2_H_4_)_2_Cl_2_ served as the catalyst. Amide 247 yields the 1,4-adduct 249 over 86% while exhibiting outstanding 1,4-selectivity. Without losing the enantiomeric excess, the 1,4-adduct 249 (98% ee) was transformed into 1,3-dithiolane 250 and subsequently reduced to produce γ,γ-diarylamide 251. Using concentrated HCl heated under reflux for about 20 hours, the amides 251 were then hydrolyzed to generate 252. The tetralone 253 was then produced by subjecting product 252 to an acid-catalyzed cyclization^179^ which resulted in a 60% yield and a 92% enantiomeric excess. Tetralone 253 is used as a preliminary step in the production of sertraline 34 (Scheme 39).^180^

Zhang *et al.* described using the asymmetric hydrogenation of γ-branched *N*-phthaloyl allyl amines with bis-phosphine-Rh complex and (*R*)-SKP, [Rh((*R*)-SKP)(cod)]SbF_6_ bearing a high biting angle, it is possible to produce enantioselective γ-lochlorogenic amine derivatives with outstanding enantio-selectivity (up to >99% ee) and decent yields.

**Scheme 40:**
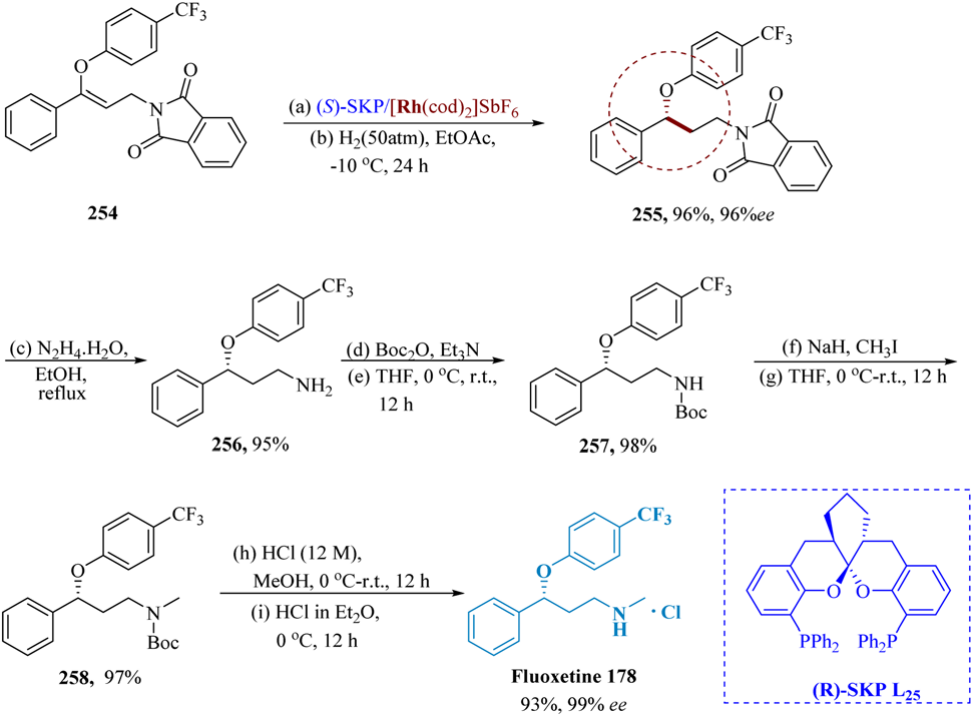
Synthesis of fluoxetine.

The intended product 255 could also be produced by hydrogenating 254 in EtOAc with L_25_ (*S*)-SKP/[Rh(cod)_2_]SbF_6_ at 50 000 S/C at room temperature and 50 atm H_2_. This method produced the intended product 255 in a good yield and with great enantioselectivity. The antidepressant medication Fluoxetine 178 was produced in various steps with >99% ee by varying the N-substituent from phthaloyl to methyl. This process yielded 83% of the desired product from the starting material 254 (Scheme 40).^181^

## 9 Iridium-catalysed reactions

**Scheme 41:**
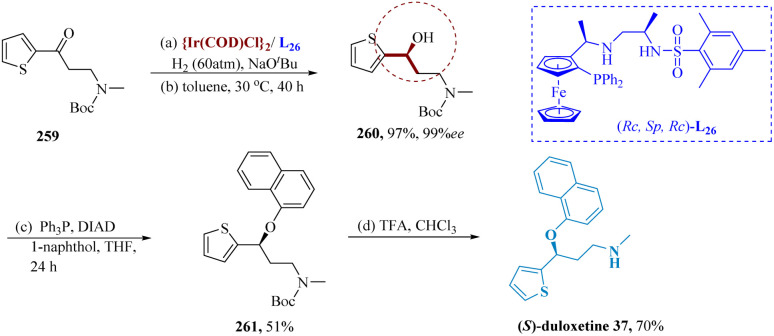
Alternative synthesis of duloxetine.

As essential intermediates in the transformation of organic material, enantiomerically enriched γ-amino alcohols, a group of widely used building blocks, plays a significant role.^182–185^ Liu *et al.* reported for (AH) of various γ-amino ketones. An effective catalytic system utilizing iridium and chiral tri-dentate ferrocene-based phosphine bearing unsymmetrical vicinal diamines were developed. Ir-(RC,SP,RC)-L_26_ catalyst efficiently hydrogenated 259, maintaining good results 97% yield and 99% ee. The respective amino alcohols result in the complete production of the desired compounds. It suggested that there was a great deal of potential for this Ir-catalyzed asymmetric conversion in industrialized applications. Additionally, *N*-Boc-*N*-methyl-(3-hydroxy)-3-(2-thienyl)propanamine 260 was obtained by Boc-protection of R-7, accompanied by Mitsunobu coupling to produce Boc-protected duloxetine 261, which was then deprotected to yield duloxetine 37 (Scheme 41).^186,187^

**Scheme 42:**
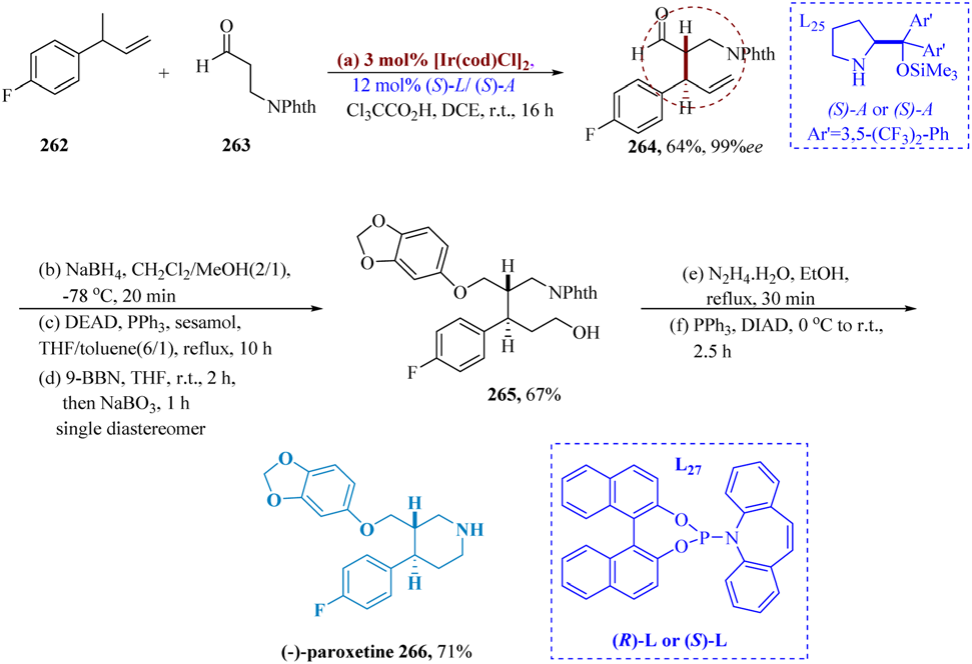
Synthesis of 3-((benzo[1,3]dioxol 5-yloxy)methyl)4-(4-fluoro phenyl)piperidine (paroxetine).

SSRIs known as paroxetine are frequently prescribed to treat panic, obsessive, and depressive disorders.^188^ Krautwald *et al.* allylated aldehyde 263 and 4-fluorophenyl vinyl carbinol 262 were allylated by Ir((*S*)-L)/(*S*)-L_27_, resulting in γ,δ-unsaturated aldehyde 264 with a yield of 64% and 6 : 1 diastereomeric ratio. After reduction to the analogous 1° alcohol, subsequently displaced to yield the respective aryl ether, separation of the diastereomers was accomplished. The terminal olefin 265 is provided by hydroboration/oxidation. (−)-Paroxetine 266 was produced through the phthalimide's cleavage and subsequent cyclization (Scheme 42).^189–191^

The neuronal uptakes of norepinephrine (NE), serotonin, or dopamine are all inhibited to an equivalent extent by the triple monoamine reuptake inhibitor diclofensine. It is a molecular derivative of tetrahydroisoquinoline (THIQ).^192,193^

**Scheme 43:**
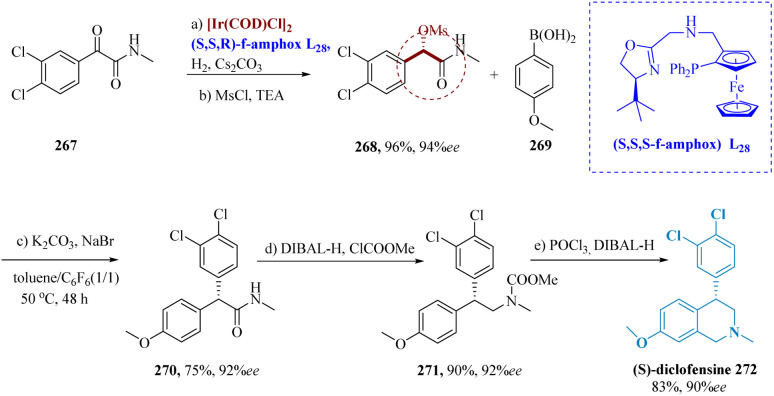
Synthesis of 4-(3,4-dichlorophenyl)7-methoxy 2-methyl-1,2,3,4tetra-hydroisoquinoline (diclofensine).

Tian *et al.* synthesized (*S*)-diclofensine Using Ir-(*S*,*S*,*R*) f-amPhox L_28_ catalyst, chiral α-α-di aryl acetamides. α-mesylates amide 268 was generated in 94% enantioselectivity and 96% by asymmetrically hydrogenating α-keto amide 267, accompanied by mesylation.^194^ The α,α-diaryl acetamide 270 was produced in 75% yield & 92% ee by stereospecific coupling of 268 and 269. (*S*)-diclofensine 272 was produced from 271 by amide reduction *via* DIBAL-H, proceeded through formylation, ring closure, as well as reduction (Scheme 43).^195–197^

## 10 Cobalt-catalysed reactions

Paroxetine, a powerful inhibitor of serotonin reuptake, is frequently recommended to cure depression, social anxiety, post-traumatic stress, OCD, and panic disorder.^198,199^

Despiau *et al.* reported cobalt-catalyzed cross-coupling reaction used to build the scaffold of the 3,4-disubstituted piperidine in a rapid way to produce (±)-paroxetine. Tri-ethyl amine (2.1 equiv.) was used as the base for the process of regioselective tosylation of diol 273, which resulted in a high yield and the generation of bromide 276. When the sesamol group was introduced with Cs_2_CO_3_ in DMF, adduct 275 was produced in 51% yield. By utilizing tetra-butyl ammonium hydroxide as a phase transfer catalyst and exposing a toluene solution of 274 and sesamol to aqueous NaOH, this yield increased to 76%. Finally, Bromo tri-phenyl phosphonium bromide was used to convert alcohol 275 into bromide 276 with a 65% yield.

**Scheme 44:**
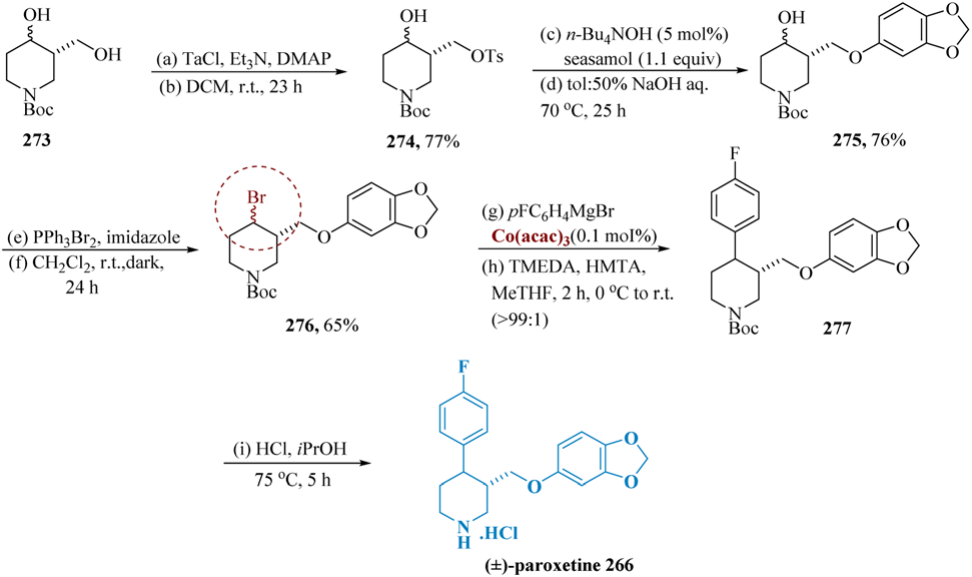
Synthesis of paroxetine.

Cross-coupling of 4-fluorophenyl magnesium bromide with bromides 276 using Co(iii) acetyl-acetonate (acac) along with TMEDA and hexamethylenetetramine (HMTA) revealed *cis*-276 producing a 16% yield of coupling product 277 with enhanced selectivity for *trans*-277. After removing the Boc protecting group and undergoing recrystallization from 2-propanol, the synthesis of (±)-paroxetine 266 was finished (Scheme 44).^200,201^

**Scheme 45:**
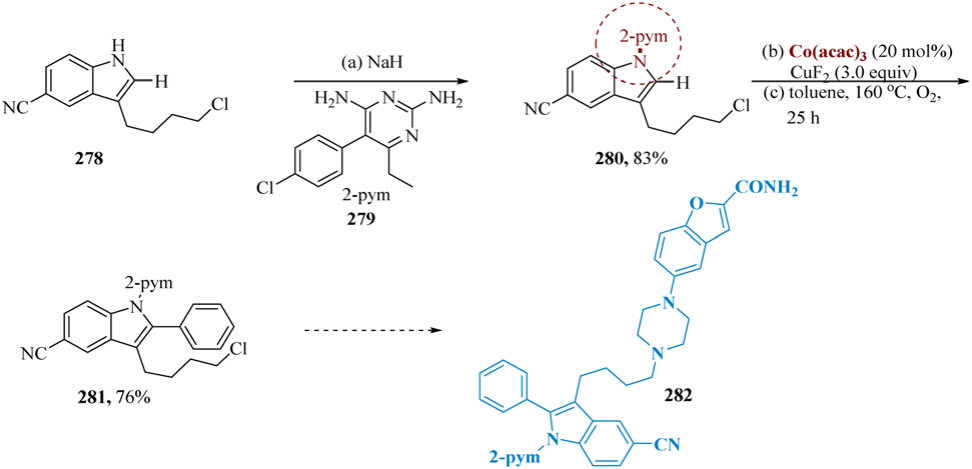
Synthesis of vilazodone derivative.

A wide range of C–H functionalizations has been achieved by cobalt catalysis with significant efficiency, and air-stable cobalt complexes abundantly present on earth have progressively emerged as stable and flexible catalysts.^202–205^ Lu *et al.* reported that this methodology has been utilized as the crucial step in the formation of the vilazodone derivative 282 from a readily accessible precursor 278. When the intermediate 280 was C–H arylated, the corresponding product 281 was produced in a 76% isolated yield and was easily transformed into a 2-arylated vilazodone derivative 282 (Scheme 45).^206,207^

Antidepressants, neuroleptics, and antiarrhythmics are only a few of the therapeutic classes represented by cationic amphiphilic drugs (CADs). Cationic amphiphilic drugs can enter cells and their organelles in their neutral, lipophilic form. These medications are effectively protonated and consequently confined in acidic cellular compartments, such as lysosomes. By making drugs more permeable to BBB in the brain, the antidepressant effect is enhanced.

**Scheme 46:**
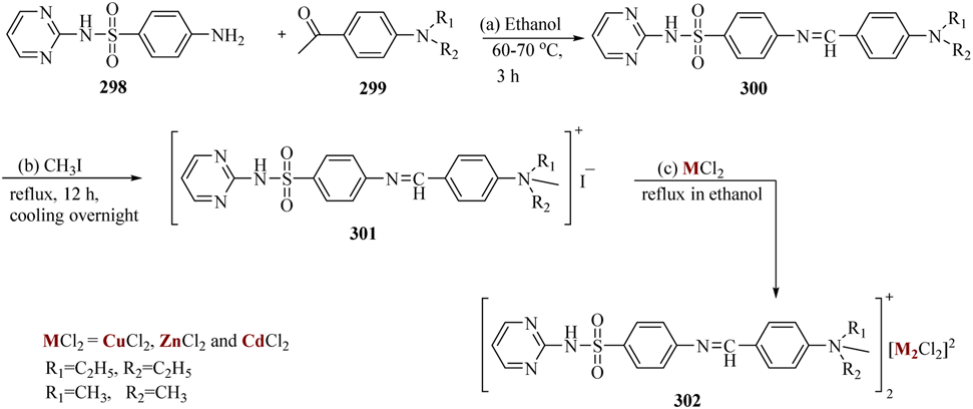
Synthesis of cationic amphiphilic drugs.

Hariprasath *et al.* reported by treating amines 298 with aromatic aldehydes 299 such as para diethyl and dimethyl amino benzaldehyde, Schiff's base of sulphadiazine was formed. By reacting with MeI, the synthesized Schiff's bases 300 were changed into their cationic amphiphilic bases 301. These bases were treated with metals such as ZnCl_2,_ CdCl_2_, and CuCl_2_, to produce metal complexes 302. Both zinc and copper metal complexes demonstrated remarkable anti-inflammatory and antidepressant properties (Scheme 46).^208,209^

**Scheme 47:**
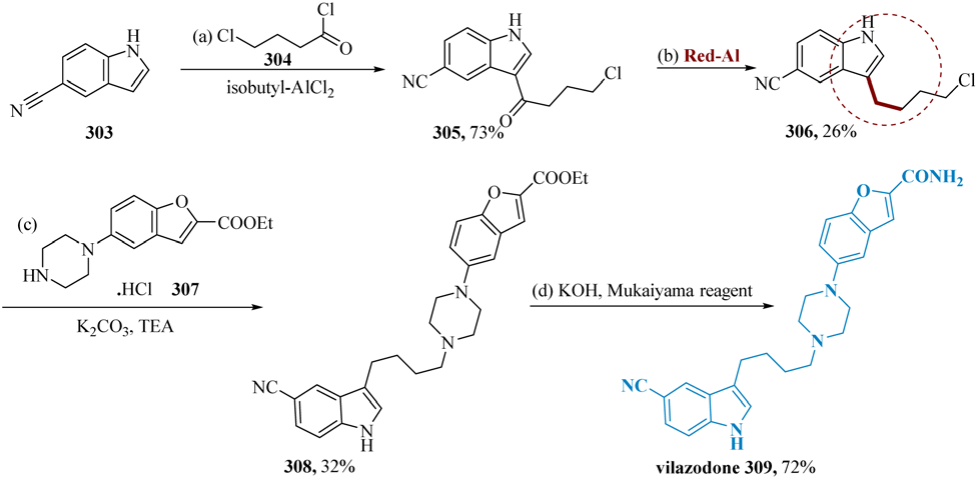
Synthesis of 5-(4-(4-(5-cyano-1*H*-indol-3-yl)butyl)piperazin-1-yl)benzo-furan 2-carboxamide (vilazodone).

Vilazodone, an SSRI as well as a partial agonist of the serotonin 5-HT1A receptor, is utilized to cure major depressive disorder (MDD).^210,211^ Jin *et al.* synthesized the product 3-(4-chloro butyl)-1*H*-indole-5-carbonitrile 306, is produced by selectively deoxygenating the keto functionality of 3-(4-chlorobutanoyl)-1*H*-indole-5-carbonitrile 305 in 26% & the resultant solid 306 is problematic to purify *via* chromatographically. Finally, the intermediate 308 is produced with a yield of 32% from 306 and the readily available commercial 307 compounds. Although 308 could be made using the Mukaiyama reagent (1-methyl-2- chloro-pyridinium iodide), vilazodone 309 must be synthesized as the target molecule (Scheme 47).^109^

**Fig. 5:**
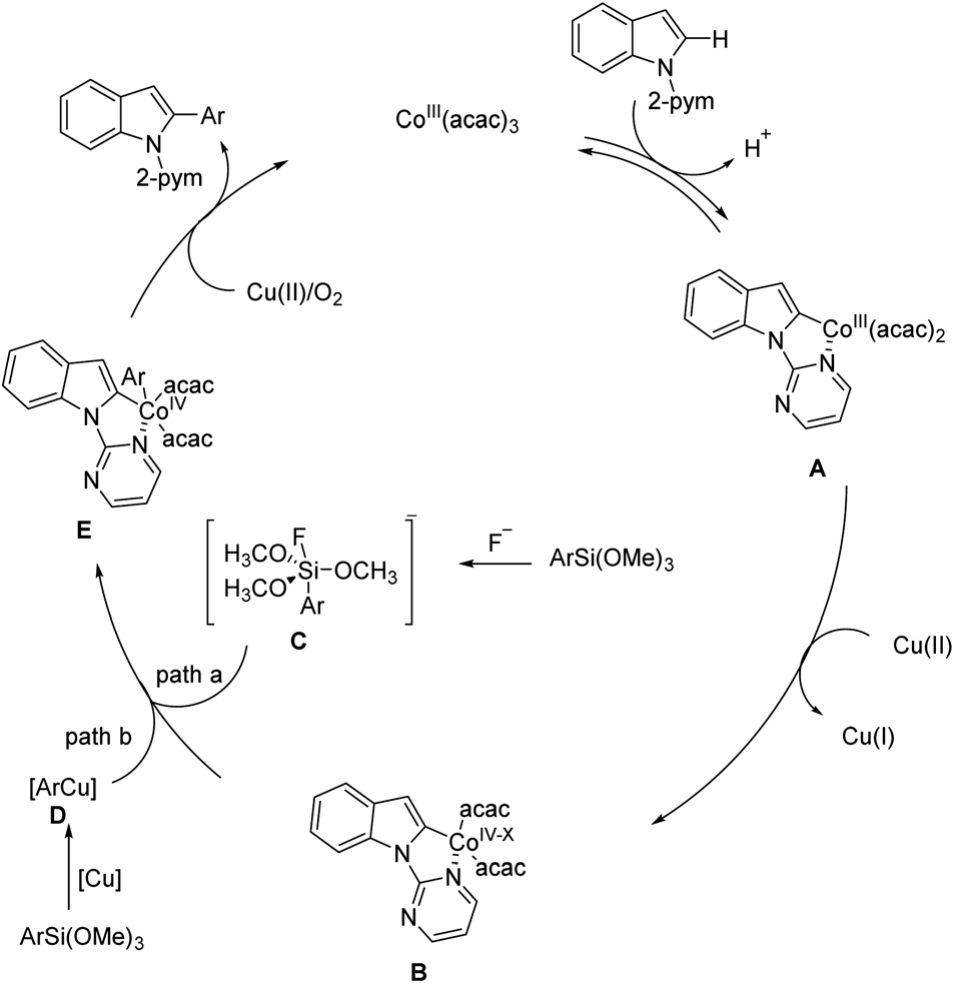
Mechanism for synthesis of vilazodone.

Initially, coordination of the nitrogen chelating group to the cobalt(iii) catalyst facilitates the reversible C–H cobaltation through a concerted metalation deprotonation (CMD) process to afford the cobaltacycle species A, which may further proceed *via* a one-electron oxidation to deliver the key cobalt(iv) intermediate B. Subsequent transmetalation with the pentavalent silicate C generated *in situ* by the fluoride ion results in the formation of cobaltacycle E (path a). Alternatively, the arylsilicate probably proceeds *via* a transmetalation reaction to give the copper–aryl species D (path b), which may render the following transfer of an aryl group to the cobalt-metal center more readily. The cobaltacycle intermediate E undergoes the oxidatively induced reductive elimination step to afford the desired arylated product and release the Co(ii) species. Finally, the Co(ii) species is re-oxidized to regenerate the Co(iii) catalyst by Cu(ii) salt or O_2_ oxidant, thus sustaining the continuity of the proposed cycle (Fig. 5).^212,213^

Agomelatine is a melatonin bio-isosteric derivative in which the naphthalene core has taken the place of the indole core.^214,215^ As a 5-HT2C receptor antagonist and agonist of MT1/MT2 melatonergic receptors, agomelatine can resynchronize disrupted circadian rhythms, alleviating sleep disorders.^216^

**Scheme 48:**
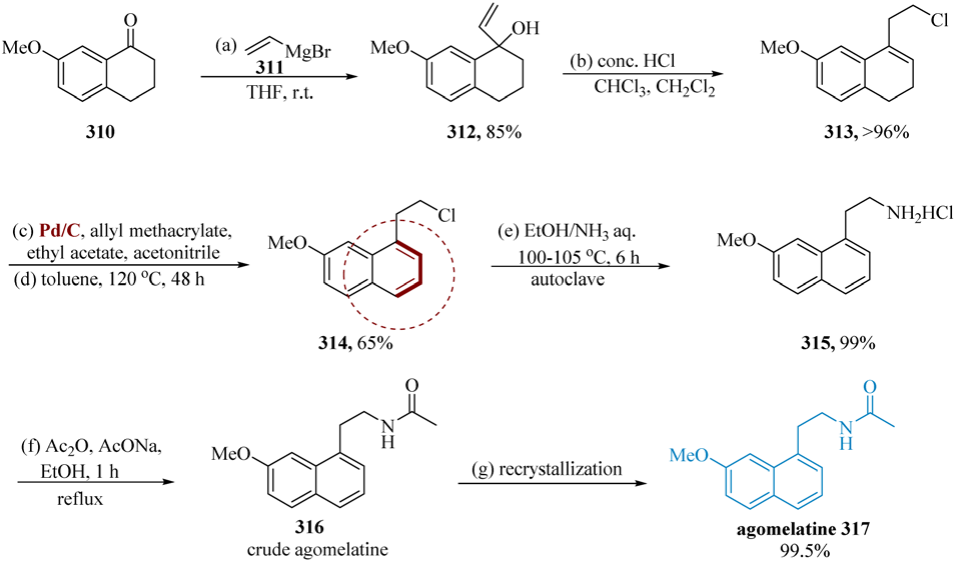
Synthesis of *N*-(2-(7-methoxy naphthalene-1-yl)ethyl)acetamide (agomelatine).

Stathakis *et al.* described that by combining vinylic organometallics with 7-methoxy-1-tetralone 310 the main building block for the synthesis of agomelatine carbinol 312 was obtained. In the next step, intermediate 313 could be obtained from carbinol 312 through allylic substitution, which is the crucial step, utilizing chlorination agents such as PCl_3,_ SOCl_2,_ or HCl as well as isomerization of the double bond. Next, chloride 313 had to be oxidized to produce the equivalent aromatic derivative 314; this was primarily done by consuming Pd in the manifestation of a hydrogen acceptor. The desired ammonium chloride 315 is produced in a decent yield by heating molecule 314 in equal volumes of EtOH and aq. NH_3_ at 100–105 °C in an auto-clave for 6 hours. By employing AcONa and Ac_2_O in EtOH heated to reflux, the acetylation of ammonium salt 315 to the analogous acetamide 316 completed the final API synthesis. After a simple re-crystallization, the pure molecule 317 was obtained with a purity of more than 99.5% (Scheme 48).^217,218^

## 11. Conclusion

Metal-catalyzed reactions have played a significant impact in the development of new pharmaceuticals, particularly antidepressants, and have transformed the field of organic synthesis. The development of more sustainable and effective routes for the synthesis of antidepressant compounds has been achieved *via* metal-catalysts. These reactions can provide the pharmaceutical industry with significant advantages, including benign reaction conditions, increased efficiency, and minimal waste output. It is estimated that metal-catalyzed reactions play a main role in the synthesis of antidepressant molecules and other therapeutics in the future owing to the ongoing advancement of novel catalysts and the continual improvement of synthetic methodologies.

## 12. Future perspectives

Developments in the field of chemistry, particularly in drug discovery and synthesis, occur rapidly. The future outlook for metal-catalyzed reactions in antidepressant molecule synthesis is promising. Researchers are likely to continue exploring new methodologies, enhancing existing processes, and addressing challenges related to scalability and regulatory requirements. Through that comprehensive survey, synthetic chemists and pharmacists develop new ideas for the derivatization of these molecules. Depressant-related diseases led to the outcome of severe mortalities in the world especially in developing countries due to various factors. So, there is a desperate need to summarize the overall possible synthetic routes through the utilization of different metals and their complexes for the synthesis of antidepressants.

## Author contributions

All Authors have equal contributions.

## Conflicts of interest

There are no conflicts to declare.

## Supplementary Material
